# Road Surface Condition Evaluation Using Imaging, LiDAR, and Multi-Grade Navigation Systems

**DOI:** 10.3390/s26144645

**Published:** 2026-07-22

**Authors:** Aser M. Eissa, Mona Hodaei, Raja Manish, Ayman Habib

**Affiliations:** Lyles School of Civil and Construction Engineering, Purdue University, West Lafayette, IN 47907, USA; eissaa@purdue.edu (A.M.E.); mhodaei@purdue.edu (M.H.); rmanish@purdue.edu (R.M.)

**Keywords:** road surface evaluation, consumer-grade accelerometers, CT-CrackSeg, LiDAR, mobile mapping systems, adaptive threshold, isolation forest

## Abstract

Road surface condition monitoring is critical for ensuring safe and efficient transportation networks. This study proposes and evaluates a framework that compares imagery-, Light Detection and Ranging (LiDAR), and accelerometer-based approaches for pavement anomaly detection. The analysis first focused on a 5-mile urban roadway segment, in which all three sensing modalities were evaluated under identical survey conditions using manually interpreted reference anomalies to compare detection accuracy, severity classification, and processing efficiency. The imagery-based Convolutional Transformer-based Crack Segmentation (CT-CrackSeg) model achieved a precision, recall, and F1-score of 88.5%, 88.5%, and 88.5%, respectively, but remained sensitive to environmental factors such as shadows, curbs, roadside features, and pavement texture variations. The LiDAR-based method achieved an F1-score of 93.0%, while the accelerometer-based Isolation Forest and Adaptive Threshold methods achieved F1-scores of 95.2% and 97.2%, respectively. These results indicate strong detection performance under the evaluated validation conditions; however, the reported precision values should be interpreted as dataset-specific rather than universal performance levels. Given the accelerometer-based approach’s strong detection performance, minimal processing time, and low deployment cost, it was further applied across a 36-mile roadway network to evaluate its scalability for network-level monitoring. Across the full route, the spatial agreement among accelerometer systems exceeded 0.91, while the agreement between the two detection methods exceeded 0.96, with 962–996 surface defects detected depending on the sensor and method. Integrating the anomaly detection results into a Potree-based web portal enabled interactive validation with geotagged imagery and point clouds, improving interpretability and diagnostic insight. Overall, the findings highlight that accelerometer-based monitoring, even with consumer-grade sensors, provides a practical, scalable, and low-cost solution for pavement evaluation, while LiDAR and imagery serve as complementary tools for detailed verification and characterization.

## 1. Introduction

**Figure 1:**
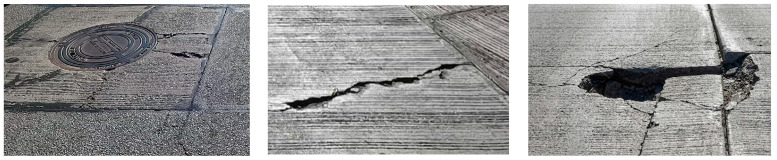
Representative road surface anomalies affecting pavement serviceability, ride comfort, and safety.

Ensuring road surface quality is essential for maintaining transportation safety, ride comfort, and pavement service life. Surface anomalies such as cracks, potholes, rutting, and depressions not only compromise driver safety but also degrade pavement performance and the overall user experience. Some examples of these irregularities are illustrated in Figure 1. Safety-related anomalies, such as deep potholes, edge failures, and abrupt discontinuities, pose the most immediate hazards to drivers and vehicles by increasing the risk of loss of control or mechanical damage. Serviceability-related anomalies, including rutting, oxidation, and surface deformation, gradually reduce the pavement’s functional performance and structural integrity, leading to accelerated deterioration and a reduced lifespan. Finally, comfort-related anomalies, such as localized unevenness or bumps, primarily affect ride quality and user comfort [1,2]. Departments of transportation (DOTs) invest substantial resources in pavement monitoring and management, yet effective large-scale assessment remains a challenge [3,4]. Traditional approaches, such as manual inspections and high-precision profiling vehicles, provide reliable measurements of pavement roughness and surface irregularities, but these methods are costly, labor-intensive, and limited in scalability [5]. As transportation infrastructure networks expand, there is a growing demand for automated, efficient, and affordable road surface evaluation technologies.

Several remote sensing approaches have been developed to address these challenges, each offering unique strengths and limitations. Imaging-based methods, which use high-resolution cameras and computer vision algorithms, provide visual interpretability and crack-level analysis [6,7]. However, these methods are highly sensitive to environmental factors such as lighting, shadows, and surface debris, which can lead to false positives and unreliable assessments [8]. Light Detection and Ranging (LiDAR) systems offer dense 3D point clouds, excelling at detecting potholes, rutting, and other major defects with high geometric precision [9,10]. Despite these advantages, LiDAR sensors are expensive, require specialized hardware, and often struggle to detect subtle or early-stage surface deterioration [11,12]. In recent years, consumer-grade inertial measurement units (IMUs) and accelerometers have emerged as cost-effective alternatives for detecting road roughness and major anomalies [13]. When mounted on regular vehicles, these systems can rapidly collect extensive data, lowering operational costs and enabling crowdsourced road monitoring. However, the sensitivity and accuracy of low-cost IMU-based methods remain underexplored, especially for fine-resolution pavement evaluation [14,15].

Although many studies focus on improving a single sensing modality, there is limited research systematically comparing these technologies under similar operational conditions. Understanding the performance trade-offs of imaging-, LiDAR-, and accelerometer-based approaches is critical for informing cost-effective pavement management strategies and guiding technology adoption by transportation agencies [16]. This study aims to fill this gap by providing a detailed evaluation of these sensing technologies, emphasizing their detection capabilities, cost implications, and operational considerations.

### Related Work

Road surface condition monitoring has been extensively studied by the transportation engineering and remote sensing communities, leading to a variety of sensing approaches and data processing strategies with different trade-offs in cost, accuracy, and scalability [17,18]. Traditional pavement condition assessment has historically relied on profilometer-based measurements of the International Roughness Index (IRI), which quantifies pavement roughness and correlates it with ride quality and serviceability. Inertial or laser profilometers mounted on specialized vehicles measure surface elevation profiles using combinations of accelerometers, laser height sensors, and distance encoders to calculate IRI. While these systems provide high accuracy and repeatability, they are costly to operate, require frequent calibration, and are impractical for frequent or large-scale road monitoring [19,20]. Consequently, research has shifted toward alternative sensing approaches that capture comparable indicators of pavement condition using lower-cost or scalable technologies such as imagery, LiDAR, and accelerometers.

Some early works used classical image processing—for example, segmentation, thresholding, and edge detection—to identify cracks and potholes in road images [21,22]. Although these approaches are simpler and more interpretable, their detection accuracy in complex real-world settings (e.g., lighting variability, shadows, and texture noise) tends to be lower than that of deep learning models. Among the latter, Heo et al. [23] introduced a Multi-Scale Feature Network (SPFPN-YOLOv4 tiny) for pothole detection in imagery and implemented a novel risk assessment framework that classified hazards based on pothole dimensions relative to tire contact patches, achieving an F1-score of 86.4%. Gorro et al. [24] extended this approach using YOLOv8 and data augmentation, including bounding box exposure and sample rotations, resulting in a model with a maximum F1-score of 0.65. While these studies demonstrate the potential of image-based deep learning models over classical image processing for road damage detection, they also highlight practical challenges such as the need for large annotated datasets, computational resources for inference, and susceptibility to environmental factors like shadows, sealants, and puddles that can cause false detections.

LiDAR-based methods have emerged as a powerful alternative, providing dense 3D point clouds and high geometric fidelity for road surface evaluation. Ravi et al. [25] developed an algorithm for a mobile mapping system equipped with a 2D profiler LiDAR. Their method incorporated scan lines and used iterative plane fitting to detect surface anomalies larger than 2 cm at highway speeds, outperforming vision- and vibration-based approaches. Talha et al. [26] combined LiDAR with YOLOv5 to detect and quantify pothole size using LiDAR-derived 2D histograms representing the spatial distribution of elevation values across the pavement surface, reporting an R^2^ value greater than 0.99 between predicted and actual pothole dimensions. These methods demonstrate LiDAR’s potential for precise and scalable pavement assessment, but they also involve high implementation costs, heavy data storage requirements, and the need for extensive preprocessing to transform raw point clouds into usable features.

In contrast, inertial sensor-based techniques focus on affordability and scalability, offering a pathway for widespread road monitoring using smartphones, vehicles, and low-cost hardware. Mednis et al. [27] investigated smartphone-based pothole detection with algorithms like Z-THRESH, Z-DIFF, STDEV(Z), and G-ZERO, demonstrating real-time detection. However, the thresholds used for vertical acceleration are not adaptive, limiting this approach to the smartphone and dataset used. Major et al. [28] used vertical acceleration data collected from connected aircraft to link IRI values to pavement joints, demonstrating inertial sensing’s versatility beyond road networks. Zang et al. [13] employed bicycle-mounted accelerometers and GPS to measure road roughness, while Celaya-Padilla et al. [29] proposed a multivariate genetic algorithm for speed bump detection, optimizing feature selection from accelerometer signals. Li et al. [30] presented the inverse pseudo-excitation method to estimate IRI from vehicle dynamic responses, improving sensitivity to changes in pavement conditions. Inertial-based approaches have been advanced by machine learning algorithms. Basavaraju et al. [31] achieved improved anomaly classification accuracy using smartphone sensors. Martinelli et al. [32] benchmarked Support Vector Machine (SVM), K-Nearest Neighbors (KNN), and decision trees for accelerometer data classification in urban and suburban environments. Arbabpour Bidgoli et al. [33] developed a low-cost monitoring platform comparable to commercial profilers. Vázquez et al. [34] studied correlations between tire–road noise, texture, and vertical acceleration. Loprencipe et al. [2] applied Root Mean Square (RMS) acceleration-IRI analysis in Rome’s urban road network. Recent research by Guerra et al. [35] expanded these methods to ATV-mounted sensors for pothole detection and IRI calculation. Singh et al. [36] highlighted the role of developing statistical models to relate the smartphone sensor acceleration to reference IRI values. In summary, these studies underscore the potential of IMU-based approaches for cost-effective and scalable pavement monitoring. However, these approaches also have limitations related to sensor noise, vehicle-dependent responses, sensitivity to speed and mounting variations, and challenges in generalizing across devices and road conditions. Vehicle dynamics are especially important in accelerometer-based road surface monitoring because the measured vertical acceleration reflects not only pavement condition but also the dynamic response of the vehicle–road interaction. Factors such as vehicle speed, suspension stiffness, tire properties, sensor mounting location, vehicle loading, and driving maneuvers can influence the amplitude and frequency content of the recorded acceleration signal. Therefore, acceleration-based anomaly detection should be interpreted as a vibration-response indicator of road surface irregularities rather than as a direct geometric measurement of pavement distress. This distinction is important when comparing accelerometer-based methods with imagery and LiDAR, which capture the visual or geometric characteristics of the pavement surface more directly.

Existing benchmark datasets have supported significant progress in automated pavement defect detection, particularly for image-based crack and road-damage analysis. Public datasets such as Crack500 and other road damage image datasets provide annotated imagery that enables training and evaluation of deep learning models for crack or pothole detection. However, these datasets are primarily image-centered and generally do not include synchronized LiDAR point clouds, GNSS/INS trajectories, vehicle acceleration measurements, or vehicle operating information. As a result, they are less suitable for evaluating the influence of vehicle dynamics or for benchmarking multimodal pavement evaluation systems. This limitation motivates the field-based comparison in this study, where imagery, LiDAR, and accelerometer measurements were collected along the same roadway segment under consistent survey conditions.

Overall, most studies evaluate these modalities independently, and there is a lack of comparative benchmarking under similar test conditions. This gap limits decision-making for transportation agencies seeking optimal monitoring solutions. To address this challenge, this research conducts a comparison of imaging, LiDAR, and inertial sensor approaches, with a particular focus on consumer-grade accelerometer systems as the lowest-cost solution. Moreover, this study evaluates detection performance, cost implications, and practical feasibility for road surface assessment.

The key contributions of the proposed research can be summarized as: (1) Development of an automated framework for detecting and classifying road surface defects using low-cost accelerometers; (2) Comparison across three sensing modalities—imagery, LiDAR, and accelerometers—to assess accuracy, scalability, and cost-effectiveness; (3) Comprehensive field validation demonstrating the feasibility of consumer-grade accelerometers for scalable pavement condition monitoring; and (4) Establishment of an interactive visualization strategy for validating anomaly detection from different modalities. The following section provides a description of the study site and the collected datasets used to illustrate and evaluate the proposed framework. It is followed by a detailed description of the image-, LiDAR-, and accelerometer-based methodologies. Section 4 then presents a comparative analysis of the image-, LiDAR-, and accelerometer-based approaches. Section 5 present the discussion, as well as the conclusions and recommendations.

## 2. Data Acquisition Systems and Dataset Description

This study employs multi-sensor, multi-platform datasets to compare the performance of different modalities in detecting roadway surface anomalies. The data were collected using Mobile Mapping Systems (MMSs) integrating LiDAR, imagery, and Global Navigation Satellite System/Inertial Navigation System (GNSS/INS)-based accelerometer systems as discussed in the following subsections. In addition, multiple grades of accelerometers were incorporated to evaluate the impact of sensor quality on anomaly detection performance. The following subsections describe the specifications and configurations of each sensing unit, along with their mounting geometry, acquisition settings, the study site and details of the acquired datasets.

### 2.1. Mobile Mapping Systems

**Figure 2:**
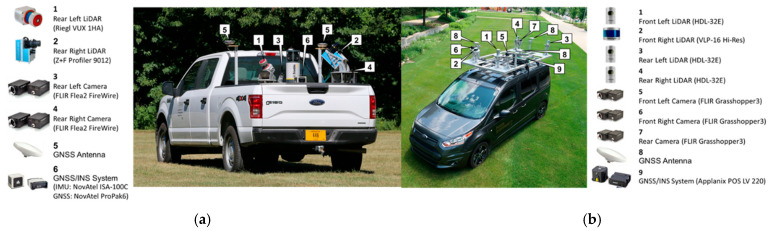
Illustration of the two PWMMS systems: (**a**) the ultra-high-accuracy system (PWMMS-UHA) and (**b**) the high-accuracy system (PWMMS-HA).

This study utilizes two mobile mapping platforms: the survey-grade Purdue Wheel-based Mobile Mapping System-Ultra High Accuracy (PWMMS-UHA) and the mapping-grade Purdue Wheel-based Mobile Mapping System-High Accuracy (PWMMS-HA), as shown in Figure 2. The PWMMS-UHA was used as the high-precision reference platform because it integrates profiler LiDAR scanners, rear-facing cameras, and a high-grade Global Navigation Satellite System/Inertial Navigation System (GNSS/INS) unit with approximately 1–2 cm positional accuracy [37,38,39,40,41]. The PWMMS-HA represents a lower-cost mapping-grade platform equipped with multi-beam LiDAR scanners, red–green–blue (RGB) cameras, and a GNSS/INS unit with approximately 2–5 cm positional accuracy [42,43,44,45]. These two systems enabled a comparison between survey-grade and mapping-grade sensing capabilities for road surface evaluation. The main specifications relevant to this study are summarized in Table A1 in Appendix A.

### 2.2. GoPro Camera-Consumer-Grade Imaging System

A forward-facing GoPro action camera (GoPro, Inc. San Mateo, CA, USA) was mounted on the front of the PWMMS-UHA system to capture high-resolution roadway imagery specifically for surface anomaly detection. The camera was tilted downward at an angle to provide a clear, unobstructed view of the pavement surface while maintaining sufficient forward visibility. This configuration maximizes roadway coverage and enhances the ability to capture fine surface details such as cracks, raveling, and potholes. Unlike the built-in cameras of the PWMMS-HA and PWMMS-UHA systems, which capture forward-facing full scene imagery primarily for mapping purposes rather than focusing specifically on the pavement surface, the dedicated GoPro setup was optimized for road surface monitoring and provided a more advantageous viewpoint for anomaly detection. The camera operated in video mode to support high-speed acquisition. The recorded frames were time-synchronized with the GNSS/INS data during post-processing using embedded time tags to ensure precise temporal alignment. Table A2 in Appendix A summarizes the main camera specifications [46].

### 2.3. Consumer-Grade Navigation System

**Figure 3:**
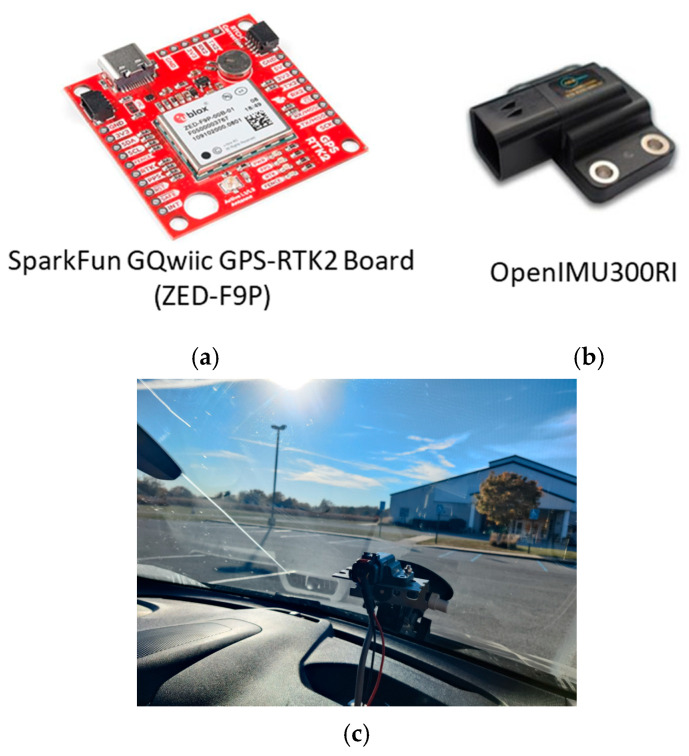
Consumer-grade GNSS/INS components and vehicle-mounted setup: (**a**) GPS-RTK2 board, (**b**) OpenIMU300RI IMU, and (**c**) integrated system on PWMMS-HA.

In addition to the GNSS/INS systems on the two mobile mapping systems, this study uses a compact, low-cost navigation system built on a SparkFun Qwiic Global Positioning System–Real-Time Kinematic 2 (GPS-RTK2) Board (ZED-F9P)(SparkFun Electronics, Niwot, CO, USA), which is based on the u-blox F9P GNSS module [47], and an OpenIMU300RI IMU(ACEINNA, Inc., Tewksbury, MA, USA) [48], both mounted together on the PWMMS-HA, as shown in Figure 3. The IMU starts in under 2 s and supports up to 100 Hz output. After post-processing, the GNSS/INS system achieved an accuracy of approximately 2–5 cm in position, 0.05° in pitch/roll, and 0.15° in heading. This is about 6 times worse than the PWMMS-HA and about 38 times worse than the PWMMS-UHA. Detailed specifications of this system are provided in Table A3 in Appendix A.

### 2.4. Study Site and Dataset Description

**Figure 4:**
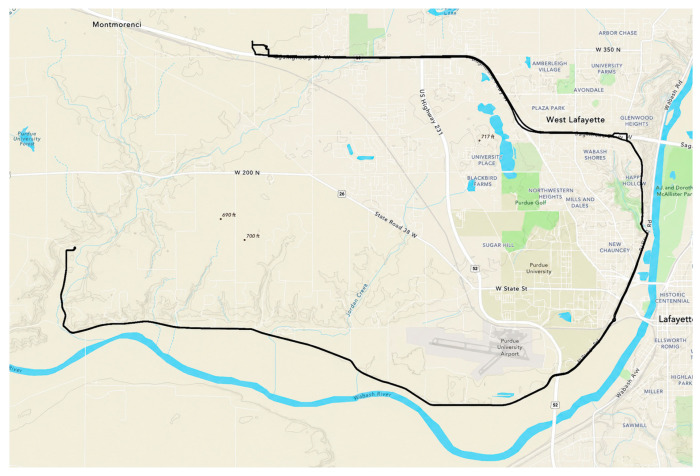
Overview of the 36-mile loop study site in West Lafayette, IN, USA, showing the dual-drive-run trajectory (in black).

**Figure 5:**
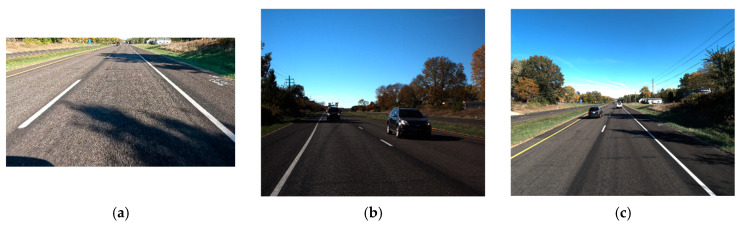
Same road section captured by: (**a**) GoPro, (**b**) PWMMS-UHA camera, and (**c**) PWMMS-HA camera.

The datasets were collected along a 36-mile closed-loop urban roadway network in West Lafayette, IN, USA, as illustrated in Figure 4. This route was selected for its representative surface conditions and realistic driving environment, encompassing sections with varied pavement types, lane configurations, and maintenance histories. Such diversity enables a comprehensive evaluation of sensing performance under realistic field conditions. The study route included both asphalt and concrete pavement sections, with asphalt representing the dominant surface type along the network. Based on the route inventory/manual review of the collected imagery, approximately 80% of the analyzed route consisted of asphalt pavement, while approximately 20% consisted of concrete pavement. Data collection was conducted under normal urban traffic conditions, including signalized intersections, stop-and-go segments, and sections with surrounding vehicles. However, the vehicles maintained consistent driving behavior where possible to reduce the influence of abrupt maneuvers on the acceleration measurements. Weather conditions were generally consistent during the survey, with dry pavement and no precipitation observed during data acquisition. These datasets include three primary sensor modalities. First, dense LiDAR point clouds were acquired using the LiDAR sensors mounted on the PWMMS-UHA, representing the three-dimensional pavement geometry along the entire route. Second, imagery was collected as georeferenced Red–Green–Blue (RGB) images from cameras integrated within the PWMMS-HA and PWMMS-UHA (approximately 15,000 images), along with forward-facing high-resolution GoPro video, from which approximately 67,000 time-tagged frames with 30 Frames Per Second (FPS) were extracted for analysis. Figure 5 compares the same road section as captured by the built-in PWMMS-HA and PWMMS-UHA cameras and the dedicated GoPro camera. Third, vertical acceleration data were recorded by the OpenIMU mounted on the PWMMS-HA, together with GNSS/INS measurements from the PWMMS-HA and PWMMS-UHA.

## 3. Methodology

This study introduces new accelerometer-based methods for classifying road surface anomalies and further compares three sensing modalities—imagery, LiDAR, and accelerometer-based systems—with respect to their effectiveness in anomaly detection and classification. The targeted pavement conditions include cracks, potholes, rutting, surface depressions, joints, bumps, and localized unevenness, representing the full range of anomalies that influence safety, serviceability, and ride comfort, as discussed in the Introduction section. These sensing technologies are selected as modern, cost-effective alternatives to traditional inertial or laser profilometer systems used for computing the IRI. To ensure a fair and comprehensive comparison, all three sensing technologies are deployed along the same roadway segment, enabling consistent data acquisition under identical conditions. The following subsections describe the processing workflows applied to each sensing modality to support anomaly detection, classification, and cross-modality comparison.

### 3.1. Imagery-Based Road Surface Condition Evaluation

**Figure 6:**
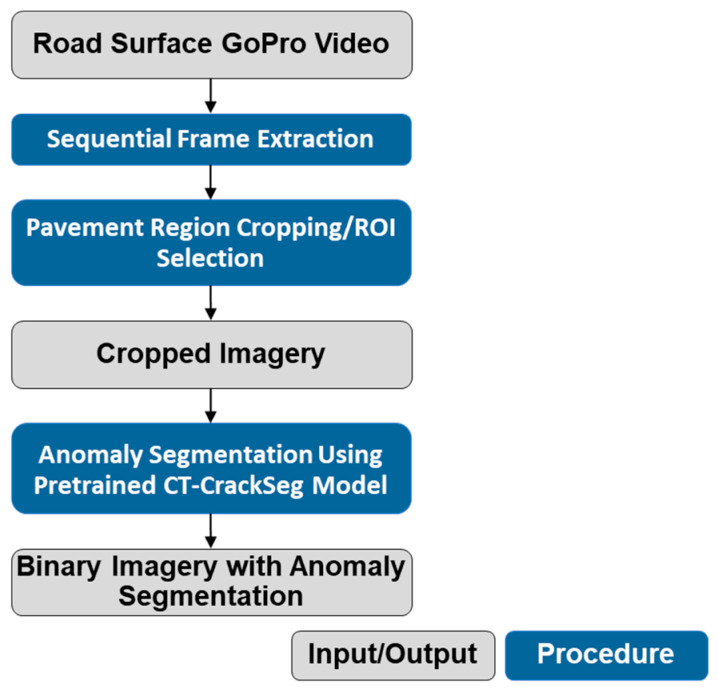
Workflow of the image-based road surface anomaly segmentation pipeline.

**Figure 7:**
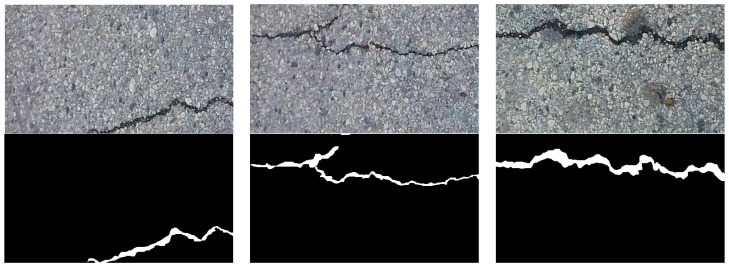
Representative samples from the Crack500 dataset used to train the CT-CrackSeg segmentation model [51].

In this study, road surface anomalies are detected and segmented from images using the approach proposed by Tao et al. [49]. Figure 6 shows the overall workflow of the image-based road surface condition evaluation, including frame extraction, preprocessing, and anomaly detection. High-resolution image frames are first extracted from a GoPro video recording. The GoPro camera is mounted at the front of the vehicle and oriented toward the road to maximize top-view coverage of the road surface. In addition, automated image processing is applied to horizontally crop the frames, retaining only the region corresponding to the road surface. This step removes most irrelevant objects, such as vehicles, sidewalks, and surrounding scenery, allowing the analysis to focus only on pavement conditions. Anomalies such as cracks and potholes are detected using the pretrained Convolutional Transformer-based Crack Segmentation (CT-CrackSeg) network [49]. This network is a lightweight transformer-based semantic segmentation model and has shown strong performance compared to other crack segmentation models on benchmark road datasets [50]. The CT-CrackSeg model was trained on the Crack500 dataset, which contains 500 high-resolution images (approximately 2000 × 1500 pixels). These images were collected using mobile phone cameras across the main campus of Temple University [51]. Figure 7 shows example images from this dataset, illustrating different types of pavement cracks used for training the model. The pretrained CT-CrackSeg model was used without additional retraining or fine-tuning on the GoPro imagery, allowing the imagery-based approach to be evaluated as an existing detection method under the same survey conditions as the LiDAR- and accelerometer-based approaches.

### 3.2. LiDAR-Based Road Surface Condition Evaluation

**Figure 8:**
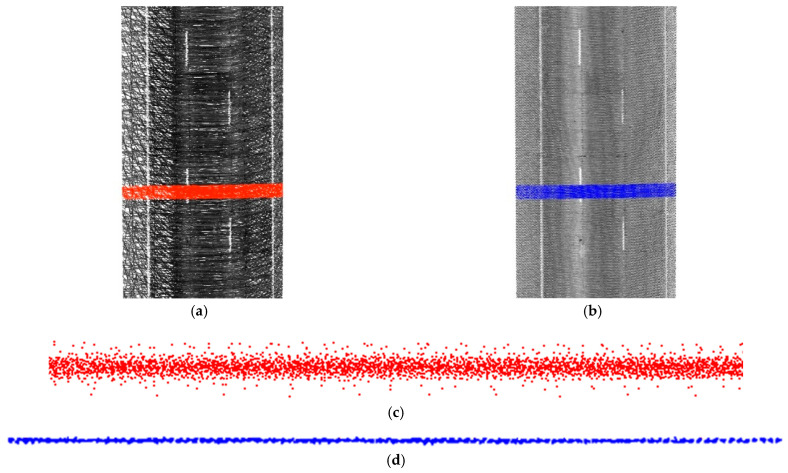
Point-cloud comparison from (**a**) mapping-grade and (**b**) survey-grade systems, with corresponding cross-sectional profiles from (**c**) mapping-grade and (**d**) survey-grade data.

**Figure 9:**
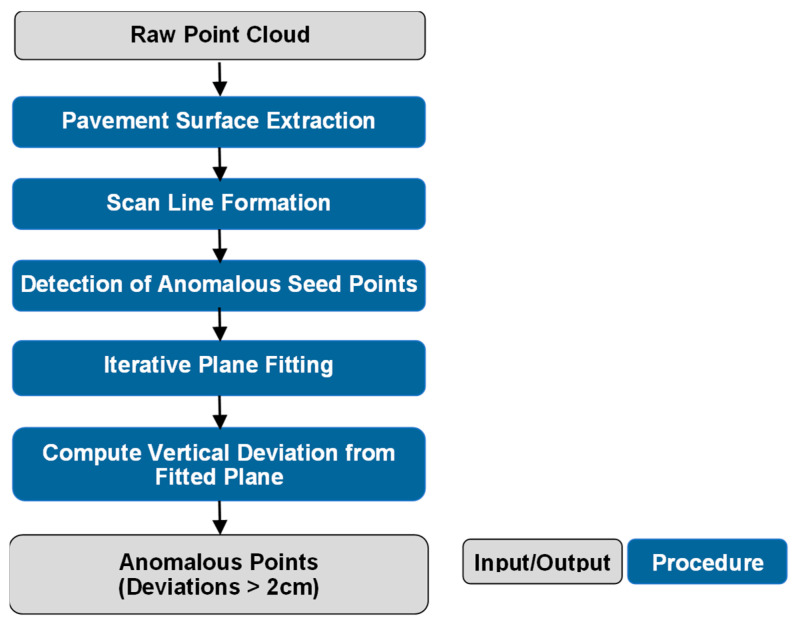
Workflow of the LiDAR-based road surface anomaly detection pipeline.

This study applies the LiDAR-based pavement distress detection developed by Ravi et al. [25]. It has demonstrated reliable performance in identifying road surface anomalies directly from mobile mapping point cloud data. As shown in Figure 8, the PWMMS-HA, a mapping-grade LiDAR system, produces noticeably noisier point clouds relative to the PWMMS-UHA, a survey-grade system. The authors emphasized that a 2D profiling LiDAR (survey-grade) is employed because it provides dense, high-precision cross-sectional measurements of the pavement surface, enabling reliable detection of surface irregularities such as cracks and potholes at highway speeds. The overall workflow of the adopted method is summarized in Figure 9.

**Figure 10:**
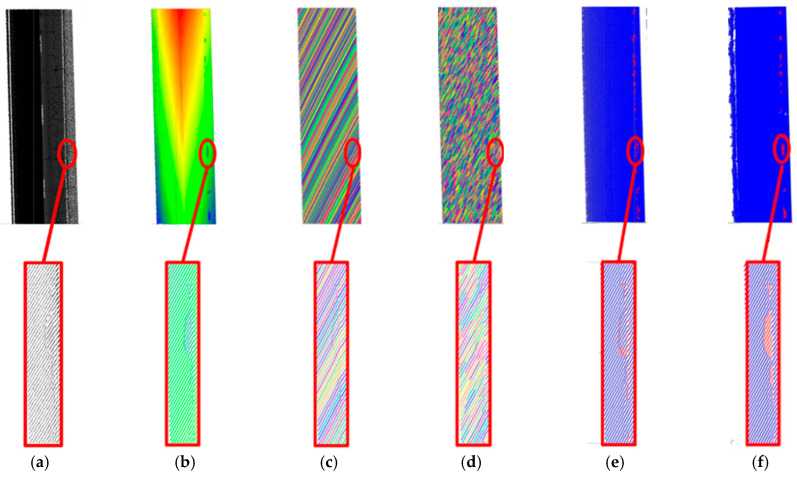
LiDAR-based anomaly detection: (**a**) intensity, (**b**) height, (**c**) scan lines, (**d**) 1 m segments, (**e**) seed points, and (**f**) final classification. Red indicates anomalies; blue indicates normal pavement.

The extracted pavement points are first organized into individual scan lines. In this context, a scan line refers to the sequence of 3D points captured during one sweep of the 2D profiler LiDAR across the pavement surface. As the vehicle moves forward, the profiler LiDAR repeatedly scans the road surface, producing a series of nearly parallel scan lines. On a smooth pavement surface, points within each scan line are expected to follow an approximately straight-line pattern, while cracks, potholes, debris, or localized discontinuities introduce sudden deviations from this expected pattern. Within each scan line, every one-meter interval is considered a segment, since road geometry can vary significantly over longer distances. For each one-meter segment, a line-fitting method is applied to identify points whose residuals from the fitted line exceed the adopted 2 cm threshold, marking them as potential anomaly seed points. To further evaluate the influence of this threshold, a threshold sensitivity analysis was conducted and is summarized in Table A4 in Appendix B. The results show that lower thresholds increase sensitivity to small surface deviations but may introduce additional false positives, while higher thresholds reduce false detections but miss more mild or localized anomalies. Therefore, the 2 cm threshold was retained as a practical balance between geometric sensitivity and false-positive control. Subsequently, a local neighborhood analysis is performed around each anomalous seed point, where iterative plane fitting is used. The complete LiDAR-based detection scheme is illustrated in Figure 10.

To quantify the severity of detected pavement defects, a depth–area-based classification method is developed in this study. After identifying anomalous points based on deviations from the fitted surface, nearby points are grouped into clusters, with each cluster representing a distinct surface defect. For each defect, two geometric parameters are evaluated, namely, the maximum depth from the fitted plane and the planimetric footprint area obtained from the convex hull of the clustered points. Each anomaly is then assigned two subscores—a depth score (S_d_) and an area score (S_a_)—where depth thresholds of 20–40 mm, 40–60 mm, and >60 mm correspond to scores of 1, 2, and 3, respectively, and area thresholds of 0.02–0.10 m^2^, 0.10–0.30 m^2^, and >0.30 m^2^ follow the same scoring scheme. The total score (S = S_d_ + S_a_) determines the overall severity level: mild (S = 2–3), moderate (S = 4), and severe (S = 5–6). Mild anomalies typically correspond to minor surface roughness or sealed cracks, moderate anomalies represent noticeable rutting or shallow potholes, and severe anomalies indicate deep potholes or significant surface deformation. This rule-based approach offers a transparent and physically interpretable framework for ranking LiDAR-detected pavement anomalies according to both their depth and spatial extent.

### 3.3. Accelerometer-Based Road Surface Condition Evaluation

**Figure 11:**
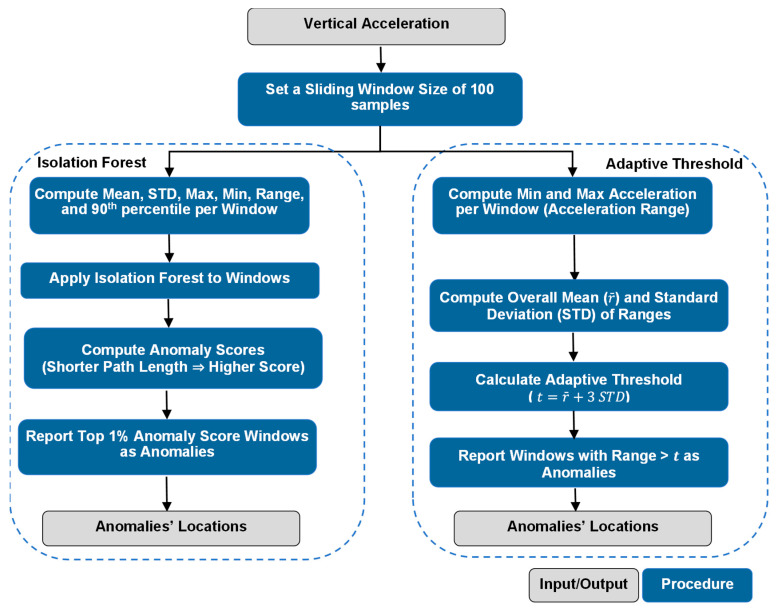
Workflow of the Isolation Forest and Adaptive Threshold methods for road surface anomaly detection.

GNSS/INS-based vertical acceleration signals are used to evaluate road surface conditions by identifying abrupt variations caused by surface irregularities and defects. Although vibration-based detection has been investigated in several previous studies, many have relied on fixed or simplified thresholding techniques that are highly sensitive to vehicle dynamics, sensor placement, and pavement texture, limiting their generalizability across different environments. To overcome these limitations, this study develops and applies two independent anomaly detection strategies, recognizing that acceleration data can generate false detections when transient noise or vehicle-induced vibrations are mistaken for surface defects. The first approach employs an unsupervised machine learning model based on the Isolation Forest algorithm [52], while the second applies a data-driven statistical approach denoted as the Adaptive Threshold method in this study. Figure 11 summarizes the workflows of both approaches, which are described in detail in the following subsections. During method development, different sliding-window sizes, Isolation Forest anomaly score cutoffs, and statistical threshold levels were evaluated to examine their influence on accelerometer-based anomaly detection. The results of this sensitivity analysis are summarized in Appendix B. Table A5 presents the sensitivity of the Isolation Forest method to different anomaly score cutoff levels. Among the evaluated cutoff levels, the top 1% cutoff provided the best balance between detection sensitivity and false-positive control and was therefore retained in the main analysis. Table A6 presents the sensitivity of the Adaptive Threshold method to different statistical threshold levels. Among the evaluated thresholds, the mean + 3STD criterion provided the best overall performance while maintaining its interpretation as a standard statistical outlier-detection threshold. In this approach, the threshold is computed from the acceleration-range distribution of each dataset rather than being imposed as a fixed absolute acceleration value. Table A7 presents the sensitivity of both accelerometer-based methods to different sliding-window sizes. Among the evaluated window sizes, the 100-sample sliding window provided the best balance between capturing localized vibration responses and reducing high-frequency noise. Based on these results, the top 1% Isolation Forest cutoff, mean + 3STD Adaptive Threshold criterion, and 100-sample sliding window were retained throughout the network-level evaluation to maintain consistency across sensors and roadway segments while avoiding case-specific tuning.

#### 3.3.1. Isolation Forest Method

The Isolation Forest [52] is an unsupervised anomaly detection algorithm designed to identify rare or irregular patterns by isolating data points rather than modeling normal behavior. The core idea is that anomalies are few and different and therefore can be isolated with fewer random splits in the data space compared to normal points. The algorithm constructs an ensemble of decision trees, where each tree is built by randomly selecting a feature and a split value. The number of splits required to isolate a data point, known as the path length, serves as a measure of its normality; shorter path lengths indicate a higher likelihood of being anomalous.

In this study, the vertical acceleration signals are first segmented into windows of 100 samples to preserve localized vibration signatures while minimizing temporal noise. For each window, statistical features such as the mean, standard deviation (STD), minimum, maximum, range, and 90th percentile are computed to form a compact feature vector that captures the overall vibration intensity and variability. These feature vectors are then analyzed using an ensemble of isolation trees, where each window receives an anomaly score derived from its average path length across all trees. Higher anomaly scores correspond to windows that deviate more significantly from the general vibration pattern of the road surface. The top 1% of windows with the highest anomaly scores are marked as anomalies, representing the most statistically isolated and irregular vibration events compared to the overall population of road segments. Finally, detected anomalies are classified into severity levels based on the standard deviation of vertical acceleration within each window. Anomalies in the top 10% (≥90th percentile) are labeled as severe, those between the 70th and 90th percentiles as moderate, and those below the 70th percentile as mild.

#### 3.3.2. Adaptive Threshold Method

The Adaptive Threshold method is a statistical approach that identifies anomalies by comparing signal characteristics to dynamically set thresholds for vertical acceleration. Similar to the Isolation Forest pipeline, vertical acceleration signals are segmented into windows of 100 samples. Instead of extracting a broad feature set, the method focuses on the min/max (range) of acceleration values within each window as the key indicator of pavement anomalies. The range captures the peak-to-valley vibration caused by surface irregularities and is particularly effective for distinguishing smooth pavement from anomalies such as potholes, cracks, or joints. The main advantage of this approach is the use of an adaptive threshold, as defined in Equation (1), which enables the method to account for variations across different datasets.

(1) $$t=\bar{r}+3STD$$

In Equation (1), *t* is the adaptive threshold, $\bar{r}$ is the mean acceleration range across all windows, and STD is the standard deviation of the vertical acceleration. Once anomalies are detected, severity classification is directly integrated into the Adaptive Threshold framework by comparing the acceleration range within each window to the computed threshold. Windows with acceleration ranges between one and two times the threshold are classified as mild anomalies, representing minor irregularities such as surface roughness or sealed cracks. Windows with acceleration ranges between two and three times the threshold are classified as moderate anomalies, typically associated with larger cracks or shallow potholes. Finally, windows with acceleration ranges greater than or equal to three times the threshold are classified as severe anomalies, corresponding to deep potholes or significant surface discontinuities.

### 3.4. Data Visualization

**Figure 12:**
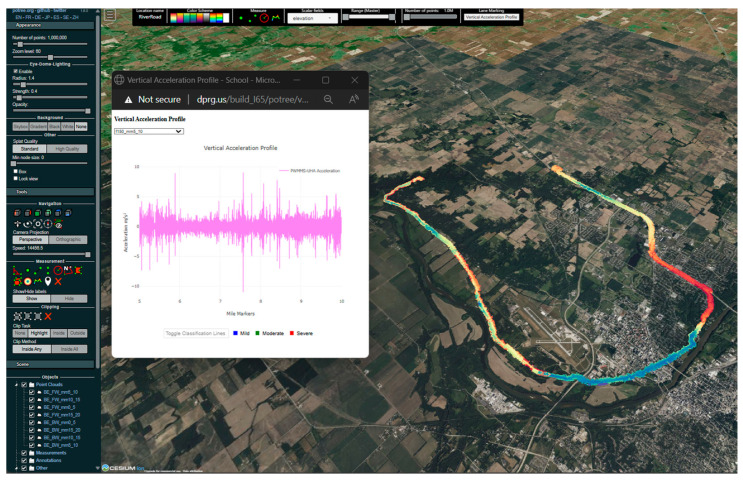
Web portal interface visualizing vertical acceleration profiles alongside the LiDAR point cloud.

**Figure 13:**
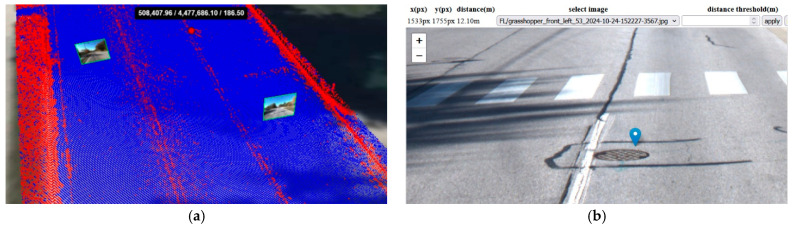
Interface of the web portal showing (**a**) the point cloud (anomalies in red) and (**b**) corresponding anomaly point back-projected onto geotagged imagery.

**Figure 14:**
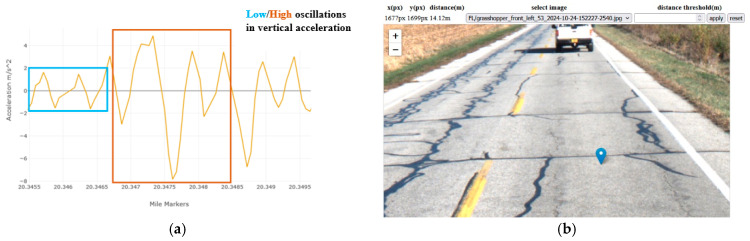
Interface of the web portal showing (**a**) the vertical acceleration profile and (**b**) corresponding anomaly point back-projected on geotagged imagery.

A reporting and visualization mechanism is developed to manage and interpret the results of the proposed anomaly detection strategies. In this research, a Potree-based web portal is employed [53,54]. Potree [55] is an open-source point cloud and imagery rendering tool based on WebGL that enables users to visualize geospatial data in a browser without the need for third-party installations or local storage of large datasets. Within the developed portal, vertical acceleration plots are integrated with georeferenced roadway imagery and point cloud data, allowing end-users to examine anomalies in the vertical acceleration signal and the corresponding imagery and point cloud. Detected anomalies can be verified by inspecting the acceleration profile along the road corridor. The portal also supports interactive functions such as selecting a point in the point cloud to display the corresponding location in the acceleration profile and using back-projection to link anomalies with roadway imagery. Users can also select a point in the acceleration profile to visualize the corresponding location in the LiDAR point cloud and imagery. These functionalities provide an efficient means to verify whether detected anomalies correspond to physical features such as cracks, joints, or potholes. Representative examples of the developed visualization system are illustrated in Figure 12, Figure 13 and Figure 14. Figure 12 presents the web portal interface showing the vertical acceleration profile as well as the LiDAR point cloud. Figure 13 demonstrates the system’s ability to visualize individual anomalies by displaying the corresponding LiDAR point cloud (with anomalies highlighted in red) and their back-projection onto geotagged imagery. Figure 14 further shows the linkage between the acceleration signal and imagery, where a selected anomaly segment from the vertical acceleration profile is spatially referenced and visualized within the road environment.

### 3.5. Comparative Evaluation Criteria

The comparative assessment of the three sensing modalities—imagery, LiDAR, and accelerometer-based systems—is structured around a comprehensive set of evaluation criteria reflecting both technical performance and practical applicability. Each method is examined in terms of cost efficiency and the ability to identify and quantify diverse pavement anomalies. Moreover, the complexity of the processing workflow and the degree of automation are also considered, recognizing that methodologies with simpler or more streamlined implementations are better suited for operational deployment. Additionally, the comparison accounts for sensitivity to external factors such as lighting, weather, and vehicle dynamics; the accuracy of anomaly localization through georeferencing; and the overall processing speed and computational efficiency of each system. To further evaluate detection quality, manually interpreted reference anomalies are generated through visual inspection of geotagged imagery, LiDAR point clouds, and the Potree-based visualization portal. The detections from each modality are compared against this reference dataset to evaluate true positives, false positives, false negatives, precision, recall, and the F1-score. Finally, the scalability of each approach is evaluated, emphasizing its potential for large-scale or continuous roadway monitoring. These criteria collectively establish a balanced framework for evaluating the trade-offs among accuracy, practicality, and cost in multi-modal road surface condition assessment.

## 4. Experimental Results and Discussion

A series of experiments was conducted to evaluate the effectiveness of three distinct approaches for pavement anomaly detection and road surface condition evaluation, namely, vision-based detection using imagery, geometry-based analysis using LiDAR point clouds, and vibration-based analysis using accelerometer data, while also comparing their relative performance under similar conditions. These methods represent different sensing modalities that vary in accuracy, cost, and scalability. The experiments were designed to assess the detection performance, classification reliability, and practical feasibility of each approach under real-world conditions. The following subsections present the experimental outcomes for each approach, outlining key findings and comparative insights.

### 4.1. Comparative Analysis of Different Modalities

To evaluate the consistency and practical applicability of the three sensing modalities—imagery-based, LiDAR-based, and accelerometer-based—a comparative analysis was performed over a 5-mile roadway segment. This section presents a direct, spatially aligned comparison of detections obtained from each sensing approach under identical survey conditions. The results were visualized through the Potree-based web portal, which enabled simultaneous inspection of LiDAR-derived anomalies, accelerometer-based detections, and their corresponding geotagged imagery. The primary objective of this comparison was to evaluate detection coverage, severity consistency, data storage, and processing efficiency among the three approaches.

Anomalies detected from imagery-, LiDAR-, and accelerometer-based approaches were georeferenced to a common roadway coordinate framework, allowing direct comparison of detection counts, severity classification, and spatial consistency. In addition to qualitative visual inspection, quantitative metrics were used to assess detection overlap and agreement across sensing modalities. To quantitatively evaluate the detection performance of each modality, a reference dataset was manually generated through visual inspection of the roadway surface using the geotagged imagery, LiDAR point cloud, and web portal. This manually interpreted reference dataset was used to identify true pavement anomalies along the 5-mile comparison segment and to evaluate true positives (TP), false positives (FP), false negatives (FN), precision, recall, and the F1-score for each approach. To establish the manually interpreted reference dataset, two members of the research team independently reviewed the 5-mile comparison segment using synchronized geotagged imagery, LiDAR point clouds, and the Potree-based visualization portal. A true anomaly was defined as a visible or geometrically identifiable pavement surface irregularity, including cracks, potholes, rutting, depressions, bumps, joints, or localized unevenness. Each candidate anomaly location was assigned a spatial reference along the roadway, and uncertain cases were reviewed using both image evidence and point-cloud geometry. Disagreements between the annotators were resolved through joint review until consensus was reached. The final reference dataset was then used consistently for all evaluated methods. A detected anomaly was counted as a true positive when it spatially corresponded to a reference anomaly within the driving lane, a false positive when no corresponding reference anomaly was present, and a false negative when a reference anomaly was missed by the method. This protocol was used to evaluate TP, FP, FN, Precision, Recall, and the F1-score for the imagery-, LiDAR-, and accelerometer-based approaches.

**Figure 15:**
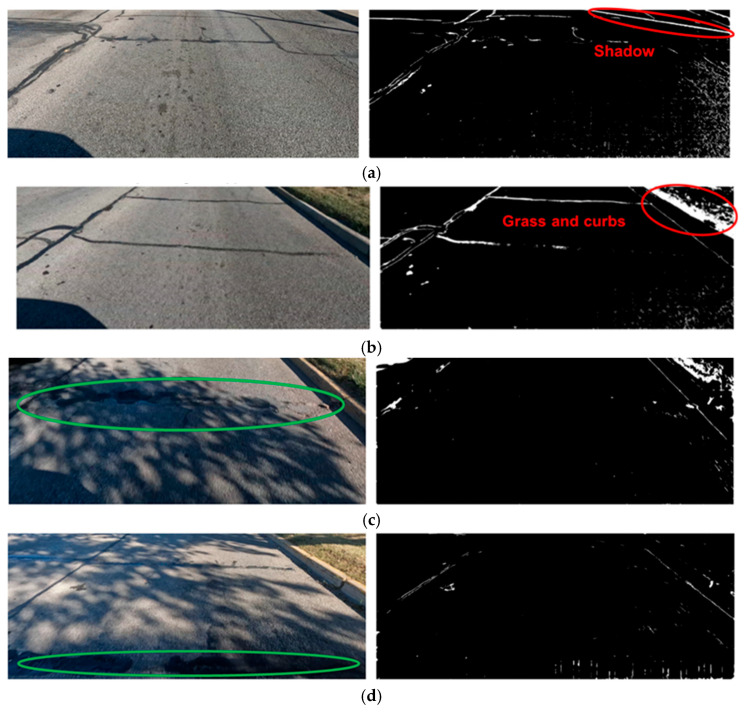
CT-CrackSeg detection errors: false positives from (**a**) sign shadows and (**b**) grass/curb regions, and false negatives for (**c**,**d**) surface deformations.

The imagery-based CT-CrackSeg model produced 130 detections, of which 115 corresponded to true positives and 15 were false positives caused mainly by shadows, curbs, roadside features, and pavement texture variations. Compared with the manually inspected reference anomalies, the imagery-based method also missed 15 anomalies (false negatives), resulting in a precision of 88.5%, recall of 88.5%, and an F1-score of 88.5%. For example, roadside curbs and shadows cast by traffic signs were misclassified as road anomalies, whereas road surface deformations were not detected as anomalies, as shown in Figure 15. Moreover, imagery-based analysis does not directly quantify anomaly severity, identifying only the presence and count of potential defects.

**Figure 16:**
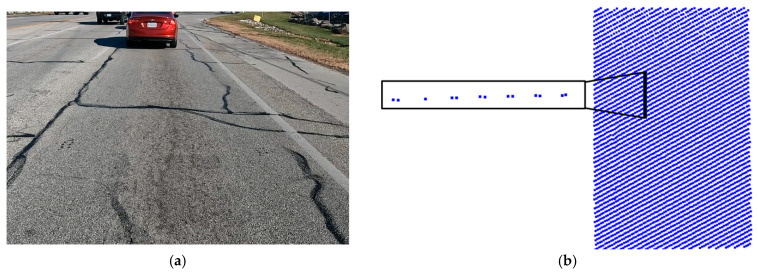

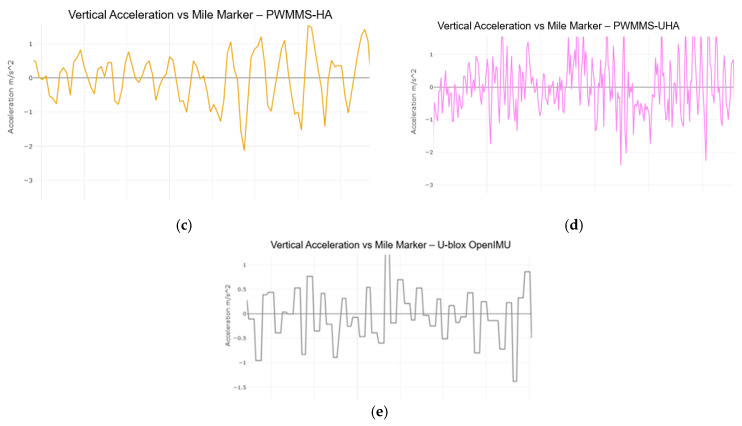
Missed anomaly example: (**a**) GoPro close-up, (**b**) classified point cloud, and vertical acceleration profiles from (**c**) PWMMS-HA, (**d**) PWMMS-UHA, and (**e**) OpenIMU. Red points indicate anomalies.

In comparison, the LiDAR-based approach detected the largest number of anomalies (208 in total) by capturing detailed geometric irregularities across multiple lanes. The LiDAR-based approach detected 113 true anomalies in the driving lane with no false positives and 17 false negatives, corresponding to a precision of 100.0%, recall of 86.9%, and an F1-score of 93.0%. In contrast, the accelerometer-based approaches—using both the Isolation Forest and Adaptive Threshold methods—produced similar detections within the driving lane. The Isolation Forest method detected 118 true anomalies with no false positives and 12 false negatives, yielding a precision of 100.0%, recall of 90.8%, and an F1-score of 95.2%. The Adaptive Threshold method achieved the highest detection performance among the evaluated approaches, with 123 true positives, no false positives, and 7 false negatives, corresponding to a precision of 100.0%, recall of 94.6%, and an F1-score of 97.2%. Figure 16 shows an example of a missed anomaly by the LiDAR- and accelerometer-based approaches corresponding to a small pavement joint. Table 1 summarizes the quantitative detection performance of the three modalities and provides a contextual comparison with recent comparable studies. These results indicate that the LiDAR- and proposed accelerometer-based methods were less prone to false positives than the imagery-based detection method, while the remaining errors were primarily associated with subtle or localized defects that were either geometrically small or did not produce a strong vehicle vibration response. Although the LiDAR- and accelerometer-based methods achieved 100% precision within the manually validated 5-mile comparison segment, this result should be interpreted cautiously. The absence of false positives was observed under the specific data collection, processing, and validation conditions of this study and should not be interpreted as a universal property of these methods. For the LiDAR-based method, the adopted 2 cm threshold helped suppress detections associated with minor pavement texture, surface roughness, and measurement noise. For the accelerometer-based methods, the anomaly selection criteria retained only strong localized vibration responses, which reduced false detections in the evaluated segment but may also have contributed to missed subtle defects. Under different roadway, traffic, vehicle-speed, suspension, sensor-mounting, pavement-texture, or validation conditions, false positives may occur. Therefore, the reported precision values represent dataset-specific validation results rather than generalizable upper-bound performance. In comparison with existing accelerometer-based studies, the proposed Isolation Forest and Adaptive Threshold methods achieved higher F1-scores than the benchmark methods, suggesting that the proposed framework provides competitive detection performance while maintaining low data volume and processing requirements. Moreover, the absence of false positives for the LiDAR- and accelerometer-based methods should be interpreted within the context of the manually validated 5-mile comparison segment rather than as a universal property of these approaches. For the LiDAR-based method, the use of the 2 cm anomaly threshold adopted from Ravi et al. [25] helped suppress detections associated with minor pavement texture, surface roughness, and measurement noise. For the accelerometer-based methods, the anomaly selection criteria retained only strong localized vibration responses, which reduced false detections in the evaluated segment. However, false positives may occur under different operating conditions, including different vehicle types, suspension systems, driving speeds, sensor mounting configurations, traffic conditions, and pavement textures.

**Figure 17:**
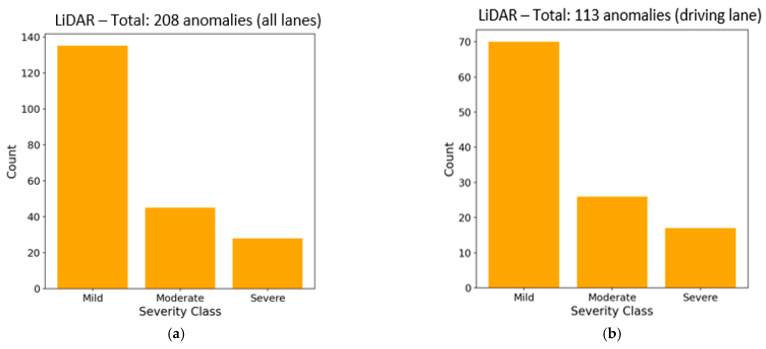

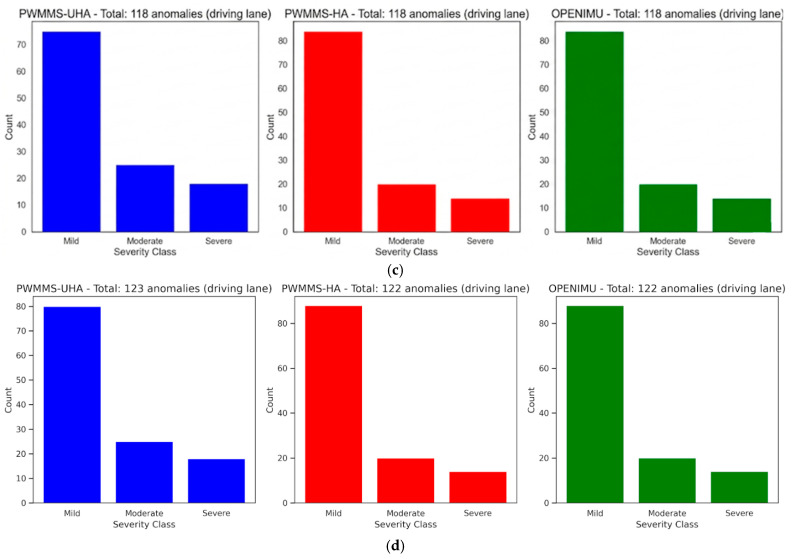
Anomaly counts and severity classes from (**a**) LiDAR in all lanes, (**b**) LiDAR in the driving lane, (**c**) Isolation Forest, and (**d**) Adaptive Threshold across the evaluated systems.

Across both modalities, most anomalies were classified as mild, followed by moderate and severe cases, as shown in Figure 17. This severity distribution is expected because mild anomalies generally represent more frequent roadway surface irregularities, such as minor roughness, sealed cracks, shallow joints, or small localized discontinuities. For the LiDAR-based approach, severity is assigned using the proposed depth–area scoring method, where each detected defect is evaluated based on its maximum depth and spatial footprint. In contrast, the accelerometer-based methods classify severity based on the magnitude of the vertical acceleration response within each detected anomaly window. Therefore, while LiDAR severity reflects the geometric characteristics of the pavement defect, accelerometer severity reflects the vehicle vibration response induced by the defect. The general consistency in severity distribution between the two modalities supports the reliability of the classification results, while differences between individual cases may be attributed to defect location, vehicle–pavement interaction, and whether the vehicle directly traversed the anomaly.

**Figure 18:**
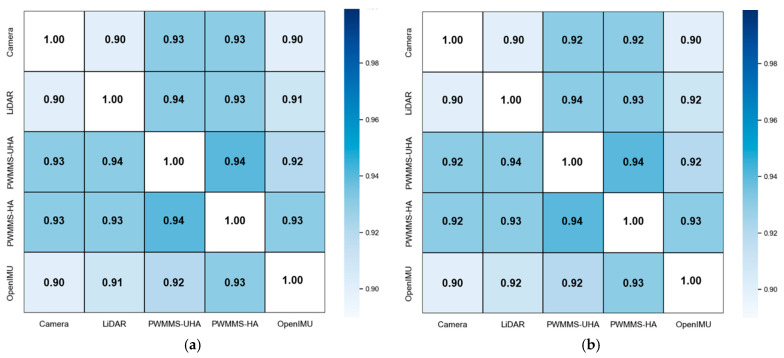
Spatial agreement of anomalies detected by camera, LiDAR, and accelerometers using (**a**) Isolation Forest and (**b**) Adaptive Threshold.

To quantitatively evaluate consistency among sensing modalities, the roadway was divided into 10 cm segments, and each segment was assigned a binary label of “1” if an anomaly was detected within the segment and “0” otherwise. Pairwise correlation coefficients were then computed to measure the degree of spatial overlap in anomaly detections between sensors. Figure 18 presents the spatial agreement among anomaly detections obtained from imagery, LiDAR, and accelerometer data, where the accelerometer-based anomalies were identified using the Isolation Forest and Adaptive Threshold methods. This figure demonstrates strong spatial agreement across sensing modalities, with correlation coefficients consistently exceeding 0.90. High agreement is observed between the LiDAR- and accelerometer-based systems, with correlation values reaching up to 0.94. Agreement between imagery-based detections and the LiDAR- or accelerometer-based approaches is slightly lower (≈ 0.90), reflecting the sensitivity of RGB-based methods to lighting conditions, shadows, and texture variations.

**Figure 19:**
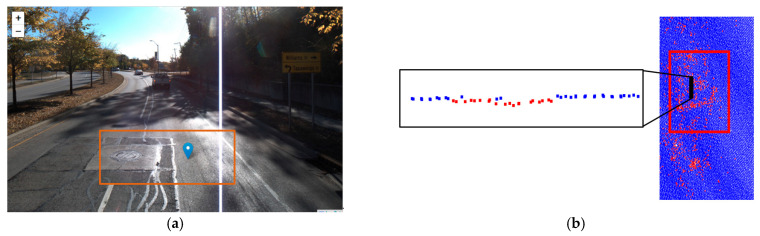

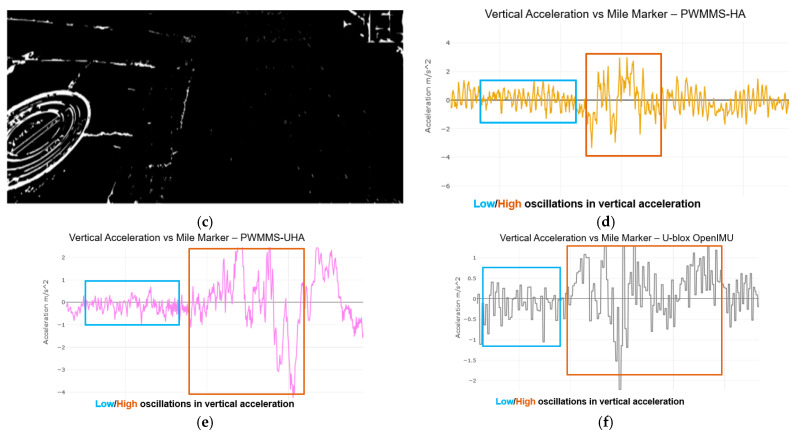
Multimodal anomaly detection at mile marker 5.70: (**a**) MMS imagery, (**b**) classified point cloud, (**c**) GoPro detection, and acceleration profiles from (**d**) PWMMS-HA, (**e**) PWMMS-UHA, and (**f**) OpenIMU. Red points and white masks indicate detected anomalies.

**Figure 20:**
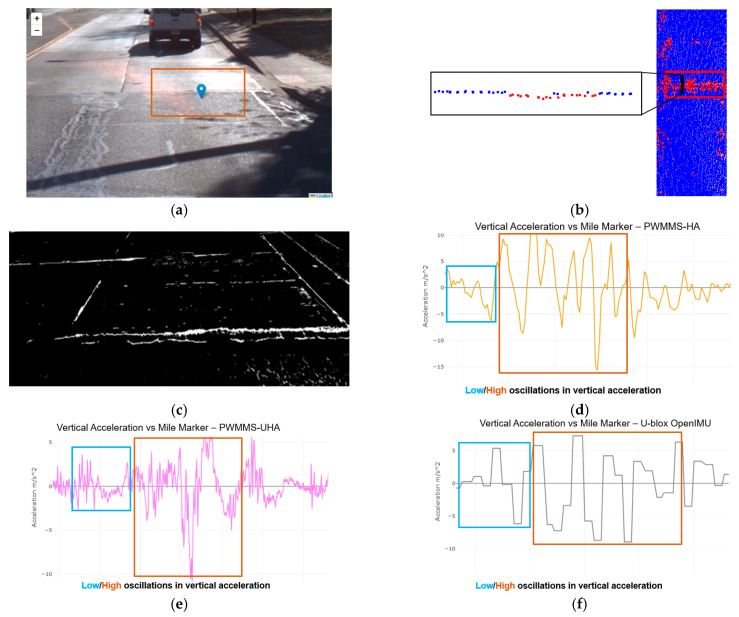
Multimodal anomaly detection at mile marker 8.34: (**a**) MMS imagery, (**b**) classified point cloud, (**c**) GoPro detection, and acceleration profiles from (**d**) PWMMS-HA, (**e**) PWMMS-UHA, and (**f**) OpenIMU. Red points and white masks indicate detected anomalies.

Despite differences in sensing principles and data characteristics, all three modalities exhibited strong consistency in detecting surface defects within the 5-mile region of interest. Figure 19 and Figure 20 illustrate two representative examples that highlight the consistency among the sensing approaches. In the first case, all three methods—LiDAR, accelerometer, and imagery—identified the same manhole feature along the driving lane. The LiDAR- and accelerometer-based approaches, with detections confirmed by both the Isolation Forest and Adaptive Threshold methods, classified this feature as a mild anomaly (e.g., Figure 19), indicating close agreement in severity and spatial alignment. The imagery-based CT-CrackSeg model also captured the same location as a potential anomaly; however, because the model relies solely on RGB information, it can only indicate the presence of a defect and cannot quantify or classify its severity. In the second case, the imagery, LiDAR, and accelerometer approaches all successfully detected a pavement joint, and both the LiDAR and accelerometer modalities categorized it as a severe anomaly, suggesting a significant geometric discontinuity and a corresponding high-magnitude vertical acceleration response.

The three modalities differ significantly in data volume, acquisition characteristics, and processing needs. LiDAR surveys generate dense three-dimensional point clouds, typically exceeding twenty gigabytes per 5-mile section, representing high-resolution pavement geometry. Imagery datasets require thousands of frames to cover the same distance. In this study, approximately 22,000 frames were extracted from the GoPro video collected along the 5-mile road segment and processed using CT-CrackSeg. To reduce redundancy among consecutive frames showing similar roadway conditions, one frame was selected from every ten consecutive frames. This sampling procedure produced a dataset of 2200 images, all of which were manually reviewed, annotated and used as the validated dataset for quantitative evaluation of the CT-CrackSeg results. In contrast, accelerometer-based data are compact, consisting only of vertical acceleration profiles and GNSS coordinates. The low data size makes accelerometers suitable for rapid transmission, lightweight data storage, and large-scale deployment.

Processing efficiency was also evaluated among the three modalities to assess their suitability for real-time or large-scale implementation. Table 2 presents the data size and the total processing time required to analyze the same 5-mile segment on a PC with an Intel Core i9-12900K processor, 64 GB of RAM, and an NVIDIA RTX A4000 GPU with 16 GB of memory. The accelerometer-based system completed processing using both approaches in approximately 2 min, followed by the imagery-based method (3 min) and the LiDAR-based method (219 min). The LiDAR-based workflow involves multiple processing stages, including point cloud reconstruction, ground filtering, and anomaly classification. Each of these steps requires high-performance computing resources and significant storage capacity, resulting in long runtimes and extensive data management efforts. Although LiDAR yields the most precise three-dimensional representation of the pavement surface, its computational and data-handling requirements limit its practicality for frequent or network-level surveys. In contrast, the imagery-based workflow is relatively faster, as it primarily relies on two-dimensional RGB data processed through a pre-trained CT-CrackSeg model. However, the model’s reliance on deep learning inference for thousands of image frames still contributes to moderate computational cost, especially when high-resolution imagery is used. The accelerometer-based approach requires minimal computational resources because it processes one-dimensional time-series data with straightforward statistical feature extraction and unsupervised anomaly detection. The Isolation Forest and Adaptive Threshold algorithms can efficiently analyze large datasets with low memory and processing overhead, enabling rapid evaluation of extended road segments.

It should be noted that the comparative evaluation was not intended to produce a formal weighted score or universal ranking of the sensing modalities. Instead, each modality was assessed across multiple criteria, including detection accuracy, severity classification, data requirements, processing time, cost, scalability, and interpretability. These criteria were considered separately because their relative importance depends on the intended application. Therefore, the overall modality recommendation was made based on application-specific trade-offs rather than a weighted ranking scheme. The cost difference between LiDAR- and accelerometer-based monitoring is also reflected in the sensor configurations used in this study. The LiDAR-based approach requires specialized mobile mapping platforms equipped with high-precision LiDAR scanners and high-grade GNSS/INS units, which increase equipment cost, deployment complexity, data storage, and processing requirements. In contrast, the accelerometer-based approach uses vertical acceleration measurements from navigation/IMU sensors, including the compact consumer-grade GNSS/INS unit, resulting in substantially smaller data volumes and shorter processing times. Therefore, accelerometer-based monitoring provides a lower-cost and more scalable option for network-level screening, while LiDAR remains more appropriate for detailed geometric characterization and verification. Overall, the comparative analysis shows that LiDAR excels in geometric information and multi-lane coverage but at high computational and cost expense, imagery provides intuitive visual interpretation but remains sensitive to environmental noise with no ability to classify the degree of the anomaly, and accelerometers achieve accurate road surface evaluation using compact data and minimal processing time. Because accelerometer-based monitoring produced reliable detections at a fraction of LiDAR’s data volume, processing time, and cost, this approach was selected for further investigation along the full 36-mile route to evaluate its scalability and network-level performance, as discussed in the following subsection.

### 4.2. Road Surface Monitoring Using GNSS/INS Acceleration Data

**Figure 21:**
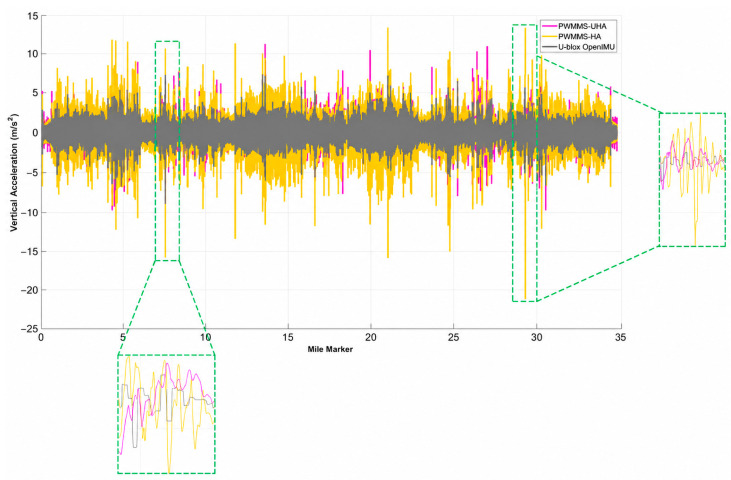
Vertical acceleration profiles across the three sensors with sample zoomed-in views.

The accelerometer-based experiments were designed to evaluate the effectiveness of survey-grade, mapping-grade, and consumer-grade sensors for pavement anomaly detection. The two anomaly detection strategies were applied to the derived vertical acceleration profiles. Figure 21 shows the vertical acceleration profiles for the used sensors. Based on the profiles, it can be interpreted that the vertical acceleration profiles from the different sensors have similar trends. The following analyses examine the performance and consistency of acceleration-based pavement anomaly detection.

**Figure 22:**
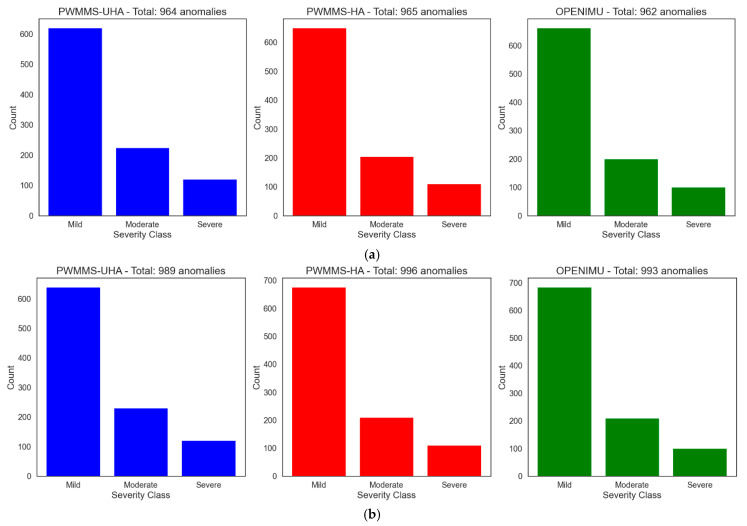
Anomaly counts and severity classes from (**a**) Isolation Forest and (**b**) Adaptive Threshold across the PWMMS-UHA, PWMMS-HA, and OpenIMU systems.

Figure 22 summarizes the total anomalies detected by the three systems and their severity using the Isolation Forest and Adaptive Threshold methods. For the Isolation Forest method, detections were consistent across sensors, with totals of 964 (PWMMS-UHA), 965 (PWMMS-HA), and 962 (ZED-FP9 Board/OpenIMU) anomalies. The majority were classified as mild, followed by moderate and severe anomalies. The Adaptive Threshold method produced slightly higher totals (989–996 anomalies) with very similar severity classifications across sensors. These findings indicate that both methods provide broadly comparable results.

**Figure 23:**
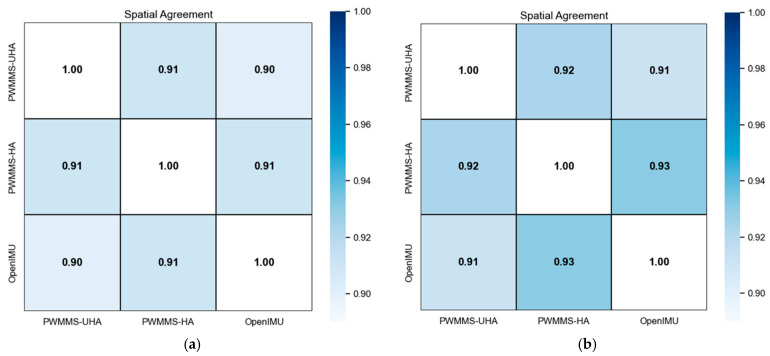
Spatial agreement of anomalies across different sensors detected by (**a**) Isolation Forest and (**b**) Adaptive Threshold.

Spatial correlation analyses were conducted to quantify the consistency of detected anomalies among the three accelerometer systems. For each detection method, the anomaly outputs from all sensors were first georeferenced and aligned along a common roadway coordinate framework (mile markers) to ensure spatial comparability. All sensor pairs exhibited strong spatial correlation, with coefficients exceeding 0.91, as shown in Figure 23, indicating a high level of agreement in identifying anomaly locations.

**Figure 24:**
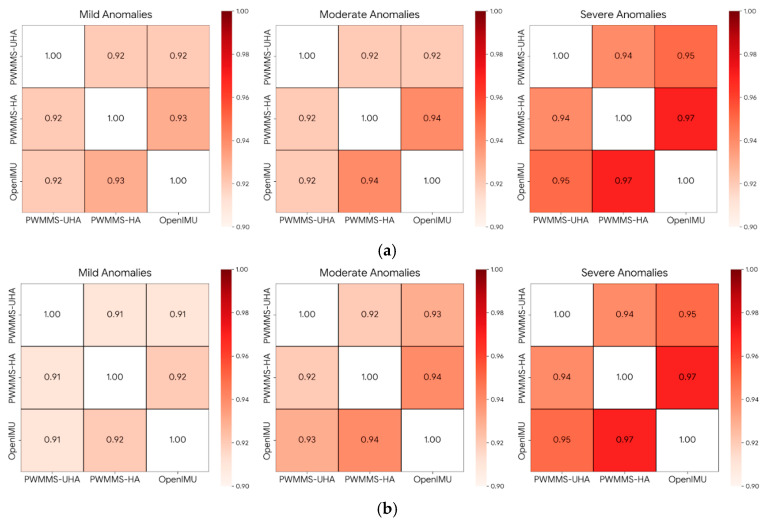
Anomaly classification agreement across different sensors detected by (**a**) Isolation Forest and (**b**) Adaptive Threshold.

For evaluating classification consistency, the same spatial segmentation was used, but with categorical labels representing anomaly severity (mild, moderate, and severe). These labels were compared to generate classification correlation matrices, as shown in Figure 24. Agreement was particularly strong for moderate and severe anomalies, which typically produce distinct vibration signatures, while mild anomalies showed slightly lower correspondence due to variations in sensor sensitivity, mounting rigidity, and vehicle suspension response. Overall, these findings confirm that the consumer-grade accelerometer has similar performance compared to professional-grade systems (mapping- and survey-grade).

To ensure the reliability of acceleration-based anomaly detection, it is essential to verify the level of agreement between the two algorithms applied in this study—Isolation Forest and Adaptive Threshold. Although both methods aim to identify surface irregularities from vertical acceleration signals, they differ fundamentally in their detection logic: the Isolation Forest relies on statistical isolation of outliers in multidimensional feature space, whereas the Adaptive Threshold uses signal range variations relative to a dynamically computed threshold. Therefore, examining their agreement helps determine whether both approaches converge toward consistent identification of pavement anomalies across varying road and sensor conditions.

**Figure 25:**
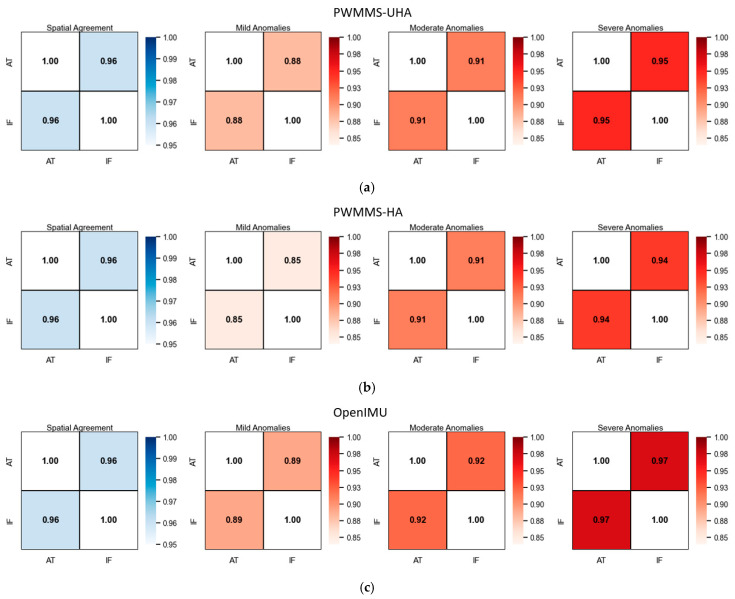
Agreement between the Isolation Forest and Adaptive Threshold methods across (**a**) the PWMMS-UHA, (**b**) PWMMS-HA, and (**c**) OpenIMU systems.

To further evaluate the consistency between the two detection algorithms, the spatial and classification agreement between the Isolation Forest and Adaptive Threshold methods was analyzed for each sensor. The corresponding agreement matrices are presented in Figure 24, which illustrates spatial correlation as well as agreement across the mild, moderate, and severe anomaly categories for the PWMMS-UHA, PWMMS-HA, and ZED-FP9 Board/OpenIMU systems. For the PWMMS-UHA sensor, both methods produced nearly identical detections, as shown in Figure 25a, demonstrating strong alignment in anomaly identification. Minor differences appeared in classification between the two methods, but overall correlation remained high. For the PWMMS-HA system, agreement was again very strong, as illustrated in Figure 25b, with both methods consistently identifying the same anomaly locations. For the ZED-FP9 Board/OpenIMU, the comparison shows that it can provide results that are competitive with the mapping- and survey-grade systems, as shown in Figure 25c. Despite higher noise levels and variability, Adaptive Threshold and Isolation Forest methods maintained correlation levels comparable to those observed with survey-grade and mapping-grade sensors. Taken together, these results confirm that both methods provide consistent outcomes across the various sensors/platforms. The strong correlation observed across methods reinforces confidence in vibration-based pavement monitoring and highlights the benefit of applying both approaches.

In addition to the correlation-based agreement analysis, McNemar’s test was conducted to statistically compare the paired binary detection outcomes of the Isolation Forest and Adaptive Threshold methods. The paired comparison showed that both methods produced strongly consistent results. The discordant cases corresponded to anomalies detected by the Adaptive Threshold method but missed by the Isolation Forest method. Using the McNemar test, the resulting *p*-value was 0.063, which is greater than the 0.05 significance level. Therefore, the difference between the two methods was not statistically significant, indicating that the Isolation Forest and Adaptive Threshold methods provide statistically comparable detection performance under the evaluated conditions.

**Figure 26:**
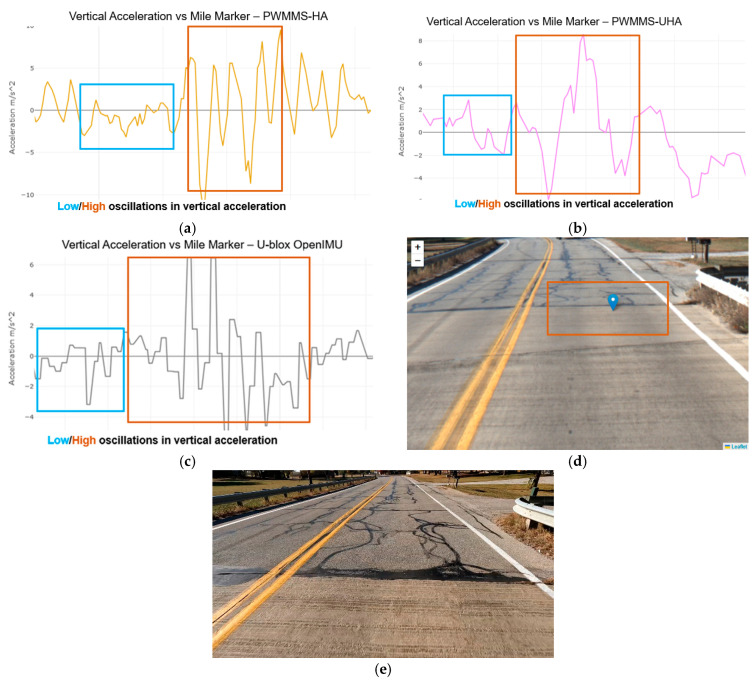
Anomaly detected at mile marker 20.14 using the Isolation Forest and Adaptive Threshold methods: acceleration profiles from (**a**) PWMMS-HA, (**b**) PWMMS-UHA, and (**c**) OpenIMU, with corresponding (**d**) MMS imagery and (**e**) GoPro close-up.

**Figure 27:**
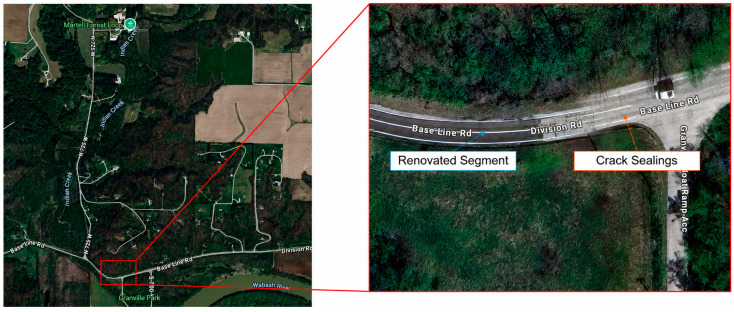
Renovated and distressed road segments along the tested dataset.

**Figure 28:**
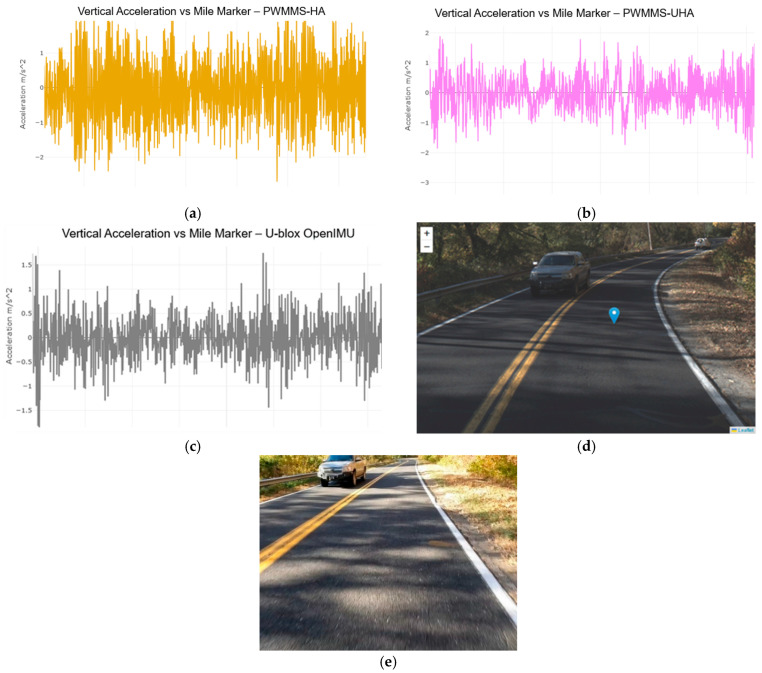
Renovated segment with no anomaly detected by the Isolation Forest or Adaptive Threshold methods: acceleration profiles from (**a**) PWMMS-HA, (**b**) PWMMS-UHA, and (**c**) OpenIMU, with corresponding (**d**) MMS imagery and (**e**) GoPro close-up.

**Figure 29:**
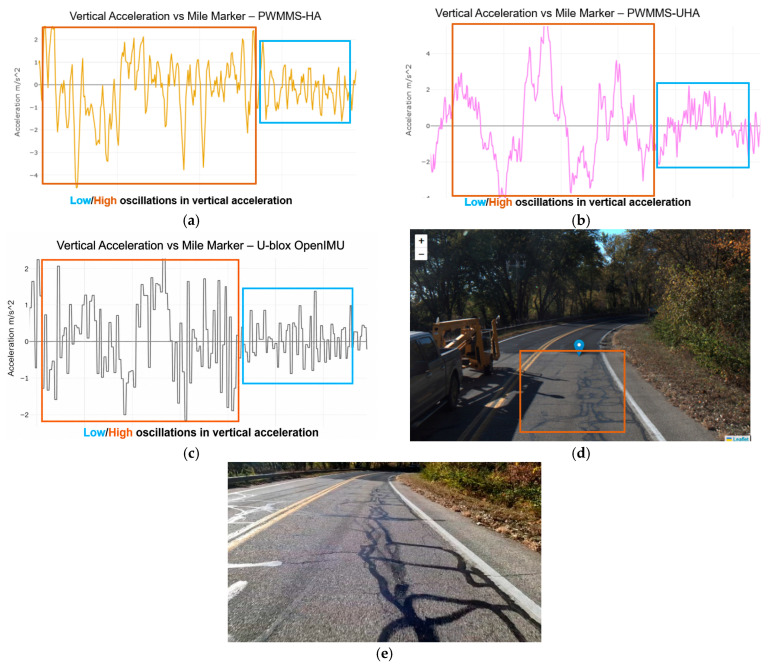
Anomaly detected at mile marker 22.41 on the old segment using the Isolation Forest and Adaptive Threshold methods: acceleration profiles from (**a**) PWMMS-HA, (**b**) PWMMS-UHA, and (**c**) OpenIMU, with corresponding (**d**) MMS imagery and (**e**) GoPro close-up.

**Figure 30:**
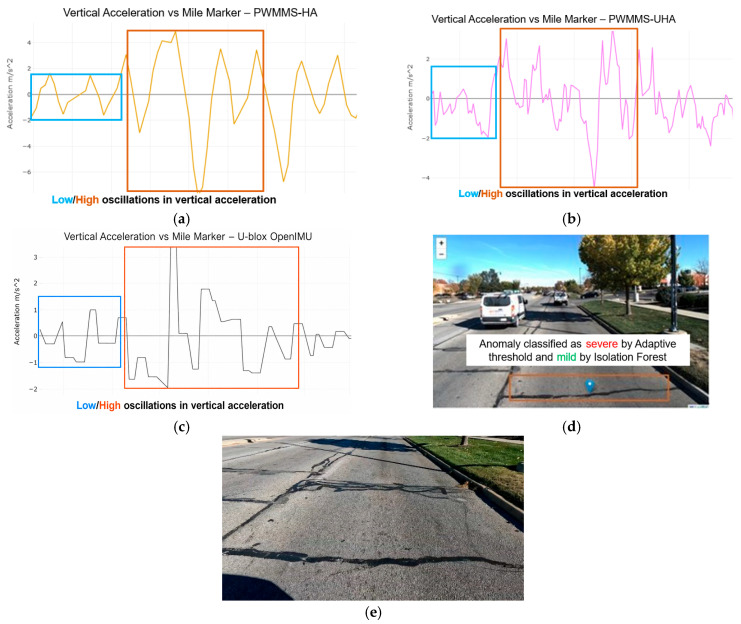
Anomaly at mile marker 5.24 classified as severe/mild by the Isolation Forest and Adaptive Threshold methods: acceleration profiles from (**a**) PWMMS-HA, (**b**) PWMMS-UHA, and (**c**) OpenIMU, with corresponding (**d**) MMS imagery and (**e**) GoPro close-up.

To provide a visual interpretation of the detection outcomes and illustrate how both methods perform under different roadway conditions, representative examples of agreement and disagreement cases are presented in Figure 26, Figure 27, Figure 28, Figure 29 and Figure 30. These examples show the corresponding vertical acceleration profiles, detected anomalies, and associated geotagged imagery across the three accelerometer systems. The Potree-based web portal played an important role in this validation process by enabling synchronized inspection of the acceleration profiles, georeferenced imagery, and LiDAR point clouds at the same roadway locations. This interactive visualization allowed detected anomalies to be verified against physical pavement features, such as joints, cracks, potholes, and surface irregularities, while also helping identify cases where differences between sensors or detection methods were caused by sensor response, vehicle dynamics, or subtle roadway conditions. Therefore, the portal served not only as a visualization tool but also as a practical validation environment for interpreting agreement and disagreement cases across the evaluated accelerometer systems.

In agreement cases, shown in Figure 26, both the Adaptive Threshold and Isolation Forest methods consistently flagged an anomaly corresponding to a road joint at the same location and assigned similar severity levels across the different sensors. Moreover, the contrast between recently renovated and older distressed roadway segments, as shown in Figure 27, offers another instructive example. On renovated pavement, both methods consistently reported no anomalies, as shown in Figure 28. However, on distressed pavement, both methods converged to detect numerous mild anomalies corresponding to crack sealings, with excellent agreement across all three accelerometer systems, as illustrated in Figure 29. This reinforces that consumer-grade accelerometers, when paired with the proposed algorithms, can capture deterioration patterns as effectively as higher-end systems. These examples confirm that the Isolation Forest and Adaptive Threshold methods are reliable, especially when anomalies produce strong vibration signatures.

In disagreement cases between the methods, illustrated in Figure 30, the Adaptive Threshold method often classified anomalies as more severe based on high acceleration ranges, while the Isolation Forest method categorized them as mild due to the impact of the nature of the data. Such discrepancies were most common on smoother roadways, where surface irregularities were subtle. These examples highlight how the two methods can be used together to increase the confidence level in detected anomalies. This variation is believed to result from differences in vehicle suspension and sensor mounting configurations. In the example shown, the anomaly corresponds to a pavement joint that was classified as severe by the PWMMS-HA sensor, mild by the OpenIMU sensor, and not detected at all by the PWMMS-UHA system.

### 4.3. Discussion

The experimental evaluation presented in this study demonstrates that each sensing modality—imaging-, LiDAR-, and accelerometer-based—offers unique strengths and limitations for roadway anomaly detection. Understanding these trade-offs is critical for guiding deployment decisions and integrating the appropriate technologies into pavement management workflows. Imaging-based approaches showed strong capability in visually identifying surface-level anomalies such as cracks and potholes, consistent with previous studies. The analysis confirmed that imagery is highly sensitive to external conditions, such as lighting, shadows, and debris, which can lead to false positives and false negatives. As indicated, image-only systems cannot reliably capture anomaly severity, which constrains their utility for quantitative assessments. In addition, although image processing is computationally light compared to LiDAR, the large number of frames that must be handled per survey increases data management effort and post-processing time when used at scale. These limitations suggest that future imaging-based implementations could benefit from more robust preprocessing strategies before anomaly detection. For example, illumination normalization, shadow detection or removal, and semantic pre-segmentation of the drivable pavement area could reduce false positives caused by shadows, curbs, vegetation, and other non-pavement regions. However, even with improved preprocessing, image-only methods may remain limited for anomalies that are expressed primarily as geometric surface deformation rather than visible texture changes. Multi-temporal image comparison could help distinguish persistent pavement defects from transient visual effects, such as shadows, debris, or temporary occlusions. In addition, integrating imagery with LiDAR and accelerometer data could improve both anomaly detection and severity estimation by combining visual evidence, geometric deformation, and vehicle vibration response. Therefore, while imagery provides valuable visual interpretation and documentation, its most effective role may be as a complementary source of information within a multi-sensor pavement evaluation framework.

LiDAR-based approaches offered precise detection of geometric anomalies and multi-lane detection, excelling at identifying structural defects such as potholes, rutting, and foreign object debris. The geometric precision of LiDAR allows detection of deviations as small as 2 cm even at highway speeds, with high repeatability. This makes LiDAR particularly effective for safety-critical applications where accuracy and reliability are paramount. Nonetheless, its major limitations are cost and computational expense. LiDAR processing for the same 5-mile segment required nearly two hours of computation, primarily due to point cloud density and 3D classification overhead. These data-intensive demands, together with the cost of hardware and storage, restrict LiDAR’s use to targeted or project-level inspections rather than routine network monitoring.

Accelerometer-based approaches—particularly those using consumer-grade sensors—emerged as a promising low-cost and computationally efficient alternative. Both the Isolation Forest and Adaptive Threshold methods demonstrated consistent detection of anomalies across survey-, mapping-, and consumer-grade accelerometers, with high spatial correlation. The simplicity of processing vertical acceleration time series enables near-real-time detection, requiring only a few minutes and representing an orders-of-magnitude reduction in computational burden compared to LiDAR. Using both detection methods together strengthens confidence in the results. The accelerometer-based approach also exhibited no false positives compared to image-based evaluation, while maintaining detection performance similar to that of more sophisticated systems. However, several limitations remain. Accuracy depends on proper sensor placement, and localized or subtle surface issues that do not induce significant vertical motion may go undetected. Furthermore, accelerometer-based anomalies can only be detected when they occur within the vehicle’s driving lane and are directly traversed by the vehicle.

To provide a more explicit failure analysis and transferability assessment, Table 3 summarizes the main failure modes and deployment considerations for each sensing modality. The evaluated methods may fail for different reasons because imagery, LiDAR, and accelerometers observe pavement condition through different physical mechanisms. Image-based detection is mainly affected by visual ambiguity, including shadows, illumination changes, curbs, vegetation, road markings, surface texture, and occlusions. LiDAR-based detection is mainly affected by geometric limitations, including shallow defects, localized defects below the adopted threshold, sparse or noisy point-cloud regions, and defects with limited vertical relief. Accelerometer-based detection is mainly affected by vehicle–road interaction, including whether the vehicle physically traverses the defect, vehicle speed, suspension characteristics, tire condition, sensor mounting, vehicle loading, and traffic-induced driving behavior. Therefore, the reported performance should be interpreted as validation under the specific vehicle, sensor configuration, roadway, traffic, and weather conditions of this study. Broader transferability requires additional validation across different vehicles, driving speeds, suspension systems, sensor placements, pavement materials, traffic conditions, weather conditions, and external multimodal datasets.

Overall, each sensing modality exhibits distinct trade-offs in accuracy, scalability, cost, and computational expense. LiDAR provides unmatched geometric information but at high processing and data-management cost; imagery offers interpretable visual outputs but requires extensive data curation; and accelerometers achieve reliable detections using lightweight data and minimal computation, making them the most scalable option for network-level assessment. Table 4 summarizes these trade-offs, highlighting the comparative advantages and constraints of imagery-, LiDAR-, and accelerometer-based approaches.

## 5. Conclusions and Recommendations for Future Work

Road surface condition evaluation is essential for maintaining safe and efficient transportation networks. To support this need, this research proposed and systematically compared three modalities for pavement anomaly detection, namely, vision-based evaluation using imaging, geometry-based analysis using LiDAR point clouds, and vibration-based monitoring using accelerometer data. Each method was evaluated in terms of detection capability, detection quality, classification reliability, data requirements, processing time, cost implications, and operational feasibility.

The comparative evaluation showed that each sensing modality provided useful but different detection capabilities. Using manually interpreted reference anomalies, the imagery-based CT-CrackSeg model achieved an F1-score of 88.5%, demonstrating the value of visual interpretability and direct identification of cracks and potholes. However, its sensitivity to shadows, curbs, roadside features, pavement texture variations, and reliance on large annotated datasets remain significant challenges. Within the manually validated 5-mile comparison segment, the LiDAR-based approach achieved an F1-score of 93.0%, while the accelerometer-based Isolation Forest and Adaptive Threshold methods achieved F1-scores of 95.2% and 97.2%, respectively. Although no false positives were observed for the LiDAR- and accelerometer-based methods in this validation segment, this result should be interpreted cautiously and should not be generalized beyond the specific dataset, survey conditions, and validation protocol used in this study. LiDAR remains valuable for detailed geometric characterization of pavement anomalies, while accelerometer-based monitoring provides a compact, low-cost, and scalable option for network-level screening. These results demonstrate that acceleration-based methods can achieve detection performance better than that of LiDAR while requiring much smaller data storage, shorter processing time, and lower deployment cost. The strong spatial agreement among survey-, mapping-, and consumer-grade accelerometers further confirms that low-cost sensors can contribute meaningfully to roadway monitoring. The use of the Adaptive Threshold and Isolation Forest methods also enhanced detection reliability by providing two independent approaches for identifying vibration-based anomalies. Together, these findings highlight that accelerometers enable scalable and low-cost network-level screening, while imaging and LiDAR can be reserved for targeted verification, visual interpretation, and detailed geometric characterization.

Future research will focus on several directions to strengthen these methodologies. For accelerometer-based detection, further work is needed to account for vehicle dynamics and sensor placement to reduce variability across platforms. Imaging-based methods require improvements under diverse environmental conditions, as well as expansion of annotated training datasets. LiDAR-based approaches would benefit from efforts to reduce acquisition and processing costs, enabling more practical use in large-scale surveys. Additional research will also explore the integration of multi-sensor fusion frameworks, advanced machine learning models for anomaly classification, and expansion of testing across a wider range of road types, traffic conditions, and environmental settings.

## Figures and Tables

**Table 1:** Quantitative detection performance of imagery-, LiDAR-, and accelerometer-based approaches compared with manually interpreted reference anomalies along the 5-mile segment.

Method	TP	FP	FN	Precision	Recall	F1-Score
Imagery—CT-CrackSeg [49]	115	15	15	88.5%	88.5%	88.5%
LiDAR [25]	113	0	17	100.0%	86.9%	93.0%
Accelerometer—Isolation Forest	118	0	12	100.0%	90.8%	95.2%
Accelerometer—Adaptive Threshold	123	0	7	100.0%	94.6%	97.2%
Accelerometer—Z THRESH [27]	120	36	10	77.0%	92.0%	83.9%
Accelerometer—SVM [32]	119	10	11	91.9%	91.9%	91.9%

**Table 2:** Data size and processing time (in minutes) for the chosen 5-mile segment by different approaches.

Modality	Imagery	LiDAR	Accelerometer
Data size	5.8 GB	22.4 GB	260 KB
Processing time	3	219	2

**Table 3:** Failure modes and transferability considerations for each sensing modality.

Modality	Main Failure Modes	Transferability Considerations
Imagery	Shadows, lighting variation, curbs, vegetation, road markings, occlusions, and pavement textures that resemble cracks	Sensitive to camera viewpoint, illumination, weather, pavement appearance, and image preprocessing
LiDAR	Shallow defects, sparse point density, and noisy point-cloud regions	Sensitive to point density, LiDAR accuracy, scan geometry, pavement texture, and the selected geometric threshold
Accelerometer	Defects outside the wheel path, low-vibration defects, speed-dependent responses, and effects of suspension, tire condition, sensor mounting, and vehicle loading	Sensitive to vehicle type, speed, suspension, sensor placement, road–vehicle interaction, traffic conditions, and pavement type

**Table 4:** Pros and cons of road surface condition evaluation systems.

Category	LiDAR-Based Approach	Imagery-Based Approach	Accelerometer-Based Approach
**Pros**	High accuracy in detecting anomalies ≥2 cm Reliable performance even at highway speeds (up to 60 mph) Works well under various lighting and weather conditions Not affected by occlusions from vehicles as image-based methods are Can cover more than one lane in a single drive	Cost-effective and easy to deploy with consumer cameras Leverages deep learning for automated detection Useful for visual interpretation and documentation	Low-cost and easily installed on any vehicle Lightweight data stream (vertical acceleration) enables rapid processing and near-real-time analysis Fewer false positives than imagery-based methods
**Cons**	Expensive hardware and setup (survey-grade MMS and LiDAR units) Requires expertise for data processing and calibration High data volume demands substantial storage and computing resources	Performance affected by lighting, shadows, and traffic occlusion Cannot measure anomaly severity	Accuracy depends on sensor placement May miss localized or subtle surface issues that do not cause significant vertical movement The vehicle must physically traverse the anomaly to detect it

## Data Availability

The data presented in this study are available on request from the corresponding author.

## Appendix A. Data Acquisition System Specifications

**Table A1:** Specifications of the GNSS/INS, cameras, and LiDAR units onboard the PWMMS-UHA and PWMMS-HA systems.

	PWMMS-UHA		PWMMS-HA	
**GNSS/INS Sensors**	NovAtel ProPak6; IMU-ISA-100C		Applanix POS LV 220	
Positional Accuracy (cm)	±1–2		±2–5	
Attitude Accuracy (Roll/Pitch) (°)	±0.003		±0.015	
Attitude Accuracy (Heading) (°)	±0.004		±0.025	
**LiDAR Sensors**	Riegl VUX 1HA	Z+F Profiler 9012	Velodyne VLP-16 Hi-Res	Velodyne HDL-32E
No. of laser beams	1	1	16	32
Pulse repetition rate (point/s)	Up to 1,000,000	Up to 1,000,000	~300,000	~695,000
Maximum range (m)	135	119	100	100
Range accuracy (cm)	±0.5	±0.2	±3	±2
**Cameras**	FLIR Flea2 FireWire		FLIR Grasshopper3	
Focal length (mm)	12		8	
Image Dimensions (pixels)	2248 × 2048		3376 × 2704	
Pixel Size (μm)	3.45		3.69	
Operating Temperature Range (°C)	0–45		0–50	

**Table A2:** Specifications of GoPro HERO10 Black action camera.

Camera Type	RGB Frame Camera
Focal Length (mm)	24.4
Sensor Dimensions (pixels)	5568 × 4872
Pixel Size (μm)	1.55
Operating Temperature Range (°C)	−10 to 45
Wavelength Range (nm)	400–700
Spectral Bands	3

**Table A3:** Specifications for the SparkFun Qwiic GPS-RTK2 Board and OpenIMU300RI IMU.

SparkFun Qwiic GPS-RTK2 Board and OpenIMU300RI IMU	
**Characteristics**	
Start-up time (s)	2
Max Output Data Rate (Hz)	100
Dimensions (mm)	65 × 66 × 27
Weight (g)	75
**INS Performance**	
Position (m)	2
Pitch/roll (°)	0.2
Heading (°)	0.5
Velocity (m/s)	0.05
**GNSS/INS Post-processed (open sky)**	
Position (m)	0.02–0.05
Pitch/roll (°)	0.05
Heading (°)	0.15

## Appendix B. Threshold and Parameter Sensitivity Analysis

**Table A4:** LiDAR threshold sensitivity analysis.

Threshold	TP	FP	FN	Precision	Recall	F1
1 cm	121	18	9	87.1%	93.1%	90.0%
**2 cm**	**113**	**0**	**17**	**100.0%**	**86.9%**	**93.0%**
3 cm	101	0	29	100.0%	77.7%	87.4%
4 cm	86	0	44	100.0%	66.2%	79.6%

**Table A5:** Detection cutoff sensitivity analysis for the Isolation Forest method.

Cutoff	TP	FP	FN	Precision	Recall	F1
Top 0.5%	96	0	34	100.0%	73.8%	85.0%
**Top 1%**	**118**	**0**	**12**	**100.0%**	**90.8%**	**95.2%**
Top 2%	124	7	6	94.7%	95.4%	95.0%
Top 5%	128	34	2	79.0%	98.5%	87.7%

**Table A6:** Detection threshold sensitivity analysis for the Adaptive Threshold method.

Threshold	TP	FP	FN	Precision	Recall	F1
Mean + 2STD	127	12	3	91.4%	97.7%	94.4%
**Mean + 3STD**	**123**	**0**	**7**	**100.0%**	**94.6%**	**97.2%**
Mean + 4STD	107	0	23	100.0%	82.3%	90.3%

**Table A7:** Sliding-window sensitivity framework for the accelerometer-based detection methods.

Window Size	Method	TP	FP	FN	Precision	Recall	F1
50	Isolation Forest	120	5	10	96.0%	92.3%	94.1%
**100**	**Isolation Forest**	**118**	**0**	**12**	**100.0%**	**90.8%**	**95.2%**
150	Isolation Forest	112	0	18	100.0%	86.2%	92.6%
200	Isolation Forest	105	0	25	100.0%	80.8%	89.4%
50	Adaptive Threshold	125	6	5	95.4%	96.2%	95.8%
**100**	**Adaptive Threshold**	**123**	**0**	**7**	**100.0%**	**94.6%**	**97.2%**
150	Adaptive Threshold	118	0	12	100.0%	90.8%	95.2%
200	Adaptive Threshold	110	0	20	100.0%	84.6%	91.7%
